# Root uptake and metabolization of *Alternaria* toxins by winter wheat plants using a hydroponic system

**DOI:** 10.1007/s12550-023-00477-3

**Published:** 2023-03-16

**Authors:** Julia Jaster-Keller, Marina E. H. Müller, Ahmed H. El-Khatib, Nicole Lorenz, Arnold Bahlmann, Ulrike Mülow-Stollin, Mirko Bunzel, Sophie Scheibenzuber, Michael Rychlik, Grit von der Waydbrink, Stefan Weigel

**Affiliations:** 1 Department for Safety in the Food Chain, German Federal Institute for Risk Assessment (BfR), Max‑Dohrn‑Str. 8‑10, 10589 Berlin, Germany; 2 Leibniz Centre for Agricultural Landscape Research (ZALF), Eberswalder Str. 84, 15374 Müncheberg, Germany; 3 Department of Food Chemistry and Phytochemistry, Institute of Applied Biosciences, Karlsruhe Institute of Technology (KIT), South Campus, Adenauerring 20 A, Karlsruhe, Germany; 4 Chair of Analytical Food Chemistry, Department of Life Science Engineering, Technical University of Munich (TUM), Maximus-von-Imhof Forum 2, 85354 Freising, Germany; 5 Current address: German Federal Office of Consumer Protection and Food Safety, Diedersdorfer Weg 1, 12277 Berlin, Germany

**Keywords:** Conjugated mycotoxins, Metabolites, QuEChERS, Validation, LC-MS/MS, HRMS

## Abstract

Fungi of the genus *Alternaria* are ubiquitous in the environment. Their mycotoxins can leach out of contaminated plants or crop debris into the soil entering the plant via the roots. We aim to evaluate the importance of this entry pathway and its contribution to the overall content of *Alternaria* toxins (ATs) in wheat plants to better understand the soil–plant-phytopathogen system. A hydroponic cultivation system was established and wheat plants were cultivated for up to two weeks under optimal climate conditions. One half of the plants was treated with a nutrient solution spiked with alternariol (AOH), alternariol monomethyl ether (AME), and tenuazonic acid (TeA), whereas the other half of the plants was cultivated without mycotoxins. Plants were harvested after 1 and 2 weeks and analyzed using a QuEChERS-based extraction and an in-house validated LC–MS/MS method for quantification of the ATs in roots, crowns, and leaves separately. ATs were taken up by the roots and transported throughout the plant up to the leaves after 1 as well as 2 weeks of cultivation with the roots showing the highest ATs levels followed by the crowns and the leaves. In addition, numerous AOH and AME conjugates like glucosides, malonyl glucosides, sulfates, and di/trihexosides were detected in different plant compartments and identified by high-resolution mass spectrometry. This is the first study demonstrating the uptake of ATs in vivo using a hydroponic system and whole wheat plants examining both the distribution of ATs within the plant compartments and the modification of ATs by the wheat plants.

**Supplementary Information:**

The online version contains supplementary material available at 10.1007/s12550-023-00477-3.

## Introduction

*Alternaria is* one of the most common fungal genera occurring ubiquitously worldwide and capable of infesting a wide variety of substrates (Escrivá et al. 2017; Lee et al. 2015). Although they are often responsible for post-harvest spoilage of agricultural products, they are important members of the rhizo- and phyllospheric microbiome and critical to plant health (Logrieco et al. 2009; Ostry 2008; Schiro et al. 2018).

Currently, more than 260 secondary metabolites are known to be produced by different *Alternaria* species growing on different plants such as vegetables and flower crops (Lou et al. 2013; Ostry 2008). They vary widely in structure and physicochemical properties and are sometimes potent mycotoxins with potentially harmful effects on human and animal health owing to their genotoxicity, cytotoxicity, and reproductive toxicity (Aichinger et al. 2020; EFSA – European Food Safety Authority 2011; Kollarova et al. 2018). Some of these *Alternaria* toxins (ATs) are also toxic to plants and associated with infection, colonization, and death of plants (Logrieco et al. 2009; Tsuge et al. 2013). Among the most common ATs are alternariol (AOH), alternariol monomethyl ether (AME), tentoxin (TEN), and tenuazonic acid (TeA) (EFSA – European Food Safety Authority 2011) which have been detected on a wide range of living host plants and agricultural products including harvested wheat grains and cereal products (Fraeyman et al. 2017; Hickert et al. 2016; López et al. 2016; Müller and Korn 2013). For these ATs, the European Food Safety Authority (EFSA) published a scientific opinion on the risks for animal and public health (EFSA – European Food Safety Authority 2011) as well as a dietary exposure assessment (EFSA et al. 2016). Recently, the European Commission recommended monitoring their presence and established indicative levels for AOH, AME, and TeA in certain food commodities (EC- European Commission 2022).

Plants are constantly in contact with many different xenobiotics, via application of manure or irrigation with polluted water, which pose a potential threat to plants (Bartha et al. 2014; Klampfl 2019). As a defense strategy, plants have developed a sophisticated detoxification system, which modifies the uptaken xenobiotics through a variety of enzymes (Coleman et al. 1997; Sandermann 1992, 1994) rendering them harmless for the plant itself (Wink 1997; Wolf et al. 1996).

Within plants, xenobiotics and/or metabolites are stored in vacuoles, cell walls or the apoplast (Coleman et al. 1997; Wink 1997). When parts of the plant are consumed by humans or animals, the substances stored therein could be released again during digestion (Malchi et al. 2014) which applies also to mycotoxins.

Mycotoxins are the most important secondary metabolites produced by fungal species which could also be present in the soil environment. Aflatoxin B_1_ (AFB1), in particular, has been detected in soils, but ochratoxin A, T-2 toxin, deoxynivalenol (DON), nivalenol (NIV), and zearalenone (ZEN) have also been detected (Beeton and Bull 1989; Fouché et al. 2020; Kenngott et al. 2022; Mortensen et al. 2003). *Alternaria* species have been found in soils as well and are part of the rhizosphere microbial community (Elmholt 2008; Hong and Pryor 2004; Thomma 2003) but data on the natural occurrence of ATs in soil are lacking so far.

A possible entry pathway of mycotoxins into the soil is the leaching of these components from infected plants or plant debris on the soil surface. *Alternaria* fungi have often been associated with cereal diseases of stem, leaves, and ears and were detected in high abundance during the ripening stages of ears (Schiro et al. 2018, 2019). Residuals from the preceding crops are contaminated with a high number of fungi and could also be a source of a long-lasting leaching of fungal components into the soil (Elmholt 2008; Gautam and Dill-Macky 2012; Schenzel et al. 2012). Heavy rainfall and runoff events can leach fungal propagules, mycotoxins, and toxins of plant origin (alkaloids, phytoestrogens) into the soil and streams (Hama et al. 2021; Hartmann et al. 2008).

Few studies on the uptake of mycotoxins by plants and crops in in vitro systems as well as in greenhouse and field experiments were performed. The incubation of seedlings of durum wheat and barley as well as tissue cultures with ZEN demonstrated its absorption by plant organs and the formation of several phase I and phase II metabolites that were extracted from roots and leaves (Kohn and Bunzel 2020; Kovalsky Paris et al. 2014; Righetti et al. 2017). Similar studies have been recently performed on biotransformation of T-2 and HT-2 toxin (Righetti et al. 2019) and DON (Righetti et al. 2020). Despite the chemically different substance classes, all mycotoxins investigated are metabolized by the plant or tissue systems used and are predominantly detected as glucosylated forms.

Biotransformation of ATs into their conjugated forms has already been described including sulfated forms of AOH and AME as well as the formation of *β*-D-glucopyranosides of AOH and D-glucopyranosides of AME. These conjugations were reported to take place in plant cells in vitro or during the fungal growth of specific *Alternaria* strains on nutrient media (Hildebrand et al. 2015; Kelman et al. 2015; Soukup et al. 2016; Walravens et al. 2016; Zwickel et al. 2018) but to our knowledge not in whole intact plants. Only few studies exist on the uptake of other genus-specific mycotoxins either by whole plants or plant cells including for example studies on the uptake of aflatoxins by green leafy vegetables (Hariprasad et al. 2013), corn seedlings (Mertz et al. 1980), groundnut seeds (Snigdha et al. 2015), and sugarcane (Hariprasad et al. 2015).

The aim of this work is to investigate the uptake of *Alternaria* toxins by roots of young wheat plants using a hydroponic system focusing on the three *Alternaria* toxins AOH, AME, and TeA. Two aspects are of particular interest here; how much of the *Alternaria* toxins AOH, AME, and TeA are incorporated into the different plant compartments in the tillering growth stage (Zadoks et al. 1974) and which plant-associated metabolites are formed during this process. The latter aspect requires a sterile system without any fungal contamination and therefore conducting the experiment with young wheat plants in a hydroponic system was preferred over young leafy vegetables or mature grains.

## Materials and methods

### Chemicals and materials

**Fig. 1:**
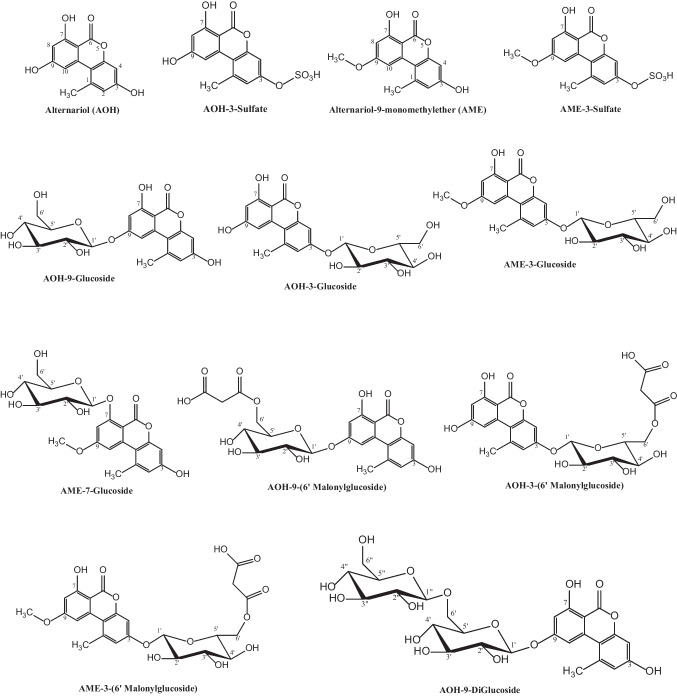
Chemical structures of the *Alternaria* toxins and conjugates reference compounds used in this study

Alternariol (AOH) with a purity of 96% was purchased from Biozol (Biozol Diagnostica Vertrieb GmbH, Eching, Germany), alternariol monomethyl ether (AME, purity 99.2%) from Biopure (Biopure, Romer Labs®, Tulln, Austria), and tenuazonic acid (TeA, purity 99.5%) from Sigma-Aldrich (Schnelldorf, Germany). The isotopically labeled derivatives ^13^C_2_-TeA (purity > 97.8%), AME-d_3_ (purity 99.9%), and AOH-d_3_ (purity 99.8%) were purchased from ASCA (Berlin, Germany). The AOH and AME conjugates AOH-3-glucoside, AOH-3-sulfate, AME-3-glucoside, and AME-3-sulfate were provided by the research group of Prof. Dr. Michael Rychlik (TU Munich – Faculty of Chemistry) (Scheibenzuber et al. 2020) and were also purchased from ASCA (Berlin, Germany) after they had become commercially available. AOH-9-glucoside, AOH-9-diglucoside, AOH-6′-malonyl-9-glucoside, AOH-6′-malonyl-3-glucoside, AME-7-glucoside, AME-6′-malonyl-3-glucoside, and AME-4′-malonyl-3-glucoside were provided by the research groups of Prof. Dr. Mirko Bunzel (KIT Karlsruhe – Department of Food Chemistry and Phytochemistry) (Hildebrand et al. 2015). The structures of these standards are shown in Fig. 1. Acetonitrile (ACN), methanol (MeOH), formic acid (FA), acetic acid (HAc), ammonium formate (NH_4_COOH), magnesium sulfate (MgSO_4_), sodium chloride (NaCl), and ethanol (Supelco, absolute) were purchased from Merck (Darmstadt, Germany). Double-deionized water was obtained using a Milli-Q system from Merck (Merck Millipore, Darmstadt, Germany).

As nutrient solution, a Hoagland solution with a final concentration of the macro-nutrients was used as follows: magnesium-sulfate heptahydrate (0.246 g/L); potassium-nitrate (0.516 g/L); ammonium-dihydrogen-phosphate (0.230 g/L); calcium-nitrate tetrahydrate (0.945 g/L). Micro-nutrients were supplemented to a final concentration as follows: iron(III)-chloride hexahydrate (2.7 µg/L); boric acid (2.86 µg/L); manganese(II)-chloride dihydrate (1.15 µg/L); zinc-sulfate heptahydrate (0.25 µg/L); copper-sulfate pentahydrate (0.08 µg/L); sodium-molybdate dihydrate (0.25 µg/L); cobalt-chloride hexahydrate (0.01 µg/L); potassium-aluminum-sulfate dodecahydrate (0.05 µg/L). The final pH of the Hoagland nutrient solution was 5.6–5.8. All chemicals were purchased from Carl Roth GmbH & Co KG (Karlsruhe, Germany).

### Design of hydroponic cultivation and wheat plant harvest

For this study, winter wheat of the cultivar ”Julius” was used. The seeds were treated by the manufacturer with a mixture of fludioxonil, tebuconazol, and difenoconazol (Landor CT, Syngenta, Germany) to exclude fungal growth in the experimental procedure. Seeds (100 g) were covered with water in a commercial jar and closed with gauze. Four jars were prepared for four germination boxes (Keimkiste “K”; Eschenfelder GmbH & Co KG, Hauenstein, Germany): two boxes with mycotoxin application and two boxes as control without mycotoxins. The jars with the wheat seeds in water were stored for 1 day at room temperature in darkness. Then the water was removed, and the seeds were rinsed with fresh water once a day and germinated in darkness for another 4 days.

The germination boxes (400 × 300 × 120 mm) consisted of a bottom section and a wash box (both food-safe plastic PE) and a stainless-steel sieve as perforated insert in the bottom part. They were surface sterilized with 70% ethanol before use. The germinated seeds (approx. 100 g) were spread evenly on the perforated insert of the germination box (Fig. S1). The bottom part of the box was filled with 4 L of Hoagland nutrient solution so that the level of the solution reached the lower edge of the sieve lying on top. A standard aquarium pump (Sera Air 275 R plus, Heinsberg, Germany) provided adequate aeration of the roots in the nutrient solution. In the next two weeks, the roots of the germs grew into the nutrient solution, and the plants and the roots grew rapidly to a height of approx. 10 cm (plants) and to a length of approx. 8 cm (roots) (Fig. S2).

The level of the nutrient solution in all germination boxes was supplemented every 2 days up to a mark of 4 L, and after 7 days, the solution was completely replaced by a new Hoagland nutrient solution of the same composition. Whenever the nutrient solution was completely changed, the containers were surface-sterilized with 70% ethanol, dried, and then refilled. Fourteen days after starting the experiment, two boxes were supplemented with a mycotoxin solution: 44.8 mg of AOH, 40 mg of AME, and 40.18 mg of TeA were dissolved in 40 mL 99% ethanol, and 250 mL deionized water was added and sonicated. Because the solution was not clear after sonication, 110 mL 99% ethanol was again added (a total of 150 mL EtOH + 250 mL deionized water) resulting in concentrations of 112 mg/L AOH, 100 mg/L AME, and 100.44 mg/L TeA.

From this solution, 120 mL was added to 4 L fresh Hoagland nutrient solution in each box (13.44 mg AOH + 12 mg AME + 12.05 mg TeA in each box). Both control boxes were supplemented with the same amount (120 mL) of diluted ethanolic solution described above. The final mycotoxin solution before application as well as the nutrient solution in each box supplemented with the mycotoxins were filled into 2 mL vials and stored at –20 °C until the LC–MS/MS analysis.

Two boxes with wheat plants (one with and one without mycotoxin supplementation) were harvested 8 days after application and the other two boxes (likewise one with and one without mycotoxin supplementation) 14 days after application. During the application period, the Hoagland nutrient solution was filled up to a mark of 4 L every second day. In the boxes which were harvested after 14 days, the nutrient solutions were completely replaced after 8 days. Mycotoxins were added in the same amount and concentration as described at the beginning of the experiment.

All germination boxes were incubated in a growth chamber (Fitotron HGC-1514; Weiss Technik UK LTD, Loughborough, UK) with a night/day air temperature of 14.5 °C/19.5 °C and a night/day air humidity of 71%/80%. The day period had a duration of 14 h. The photosynthetic active radiation was 450 µE/(m^2^s). At harvest, the sieve with the plants was lifted out of the box, and the roots were washed twice for 30 s each in a separate wash box with sterile deionized water and then placed on filter paper for 10 min to drain. After this, a sharp knife was used to cut off the roots just below the perforated insert. Each box was divided into 6 roughly equal subsamples for this purpose (Fig. S3), which were separated with string before cutting. The green above-ground plant (leaves) was then also cut off with a knife just above the perforated insert, also divided into the 6 subsamples. The then remaining crowns were removed with tweezers from the holes of the sieve and also harvested in the same 6 parts. The fresh weights of roots, leaves and crowns were determined from each of the 6 subsamples.

To examine the possibility of bacterial or fungal infections in the solution in all 4 boxes, samples of the Hoagland nutrient solution (3 × 1 mL as triplicates) were spread-plated onto nutrient media at the end of each 8- and 14-day growth period. The samples were incubated for the detection of fungal growth on potato-dextrose-agar (Merck, Heidelberg, Germany) for 5 days at 25 °C. Standard I nutrient agar (Roth, Karlsruhe, Germany) was used for the detection of bacteria. These petri dishes were incubated for 5–7 days at 30 °C.

### Preparation and extraction

The harvested and partitioned wheat samples were stored at –18 °C. Three of each of the six subsamples were combined, one of these two samples was analyzed, and the other was the retain sample. Samples were freeze-dried in a Beta 1–8 LDplus freeze dryer (CHRIST, Osterode am Harz, Germany) for 28 h. The dried samples were weighed and subsequently ground to a particle size < 0.5 mm (ring sieve pore size: 0.5 mm) using an ultra-centrifugal mill ZM200 (Retsch, Haan, Germany). Residual water content was determined with 1000 mg of each sample using the Sartorius MA30 moisture analyzer (MA30, Sartorius Corp. NY, USA). Milled samples were stored vacuum-packed at –18 °C until further analysis. Homogenized dry plant powder (1 g) was weighed in duplicates into 50 mL polypropylene (PP) conical tubes and extracted using 20 mL of ACN/water (50/50, v/v) containing 1% FA. Subsequently, the samples were shaken for 30 min (Multi Reax, Heidolph Instruments GmbH & Co. KG, Schwabach, Germany) and centrifuged for 10 min at 3500 × g (Heraeus Megafuge 16, Thermo Fisher Scientific, Waltham, USA) at room temperature. The supernatant was transferred into a new 50 mL PP conical tube. Phase separation of organic and water phase was achieved by adding 4 g anhydrous MgSO_4_ and 1 g NaCl followed by immediate shaking for 30 s (VXR B, IKA, Stauffen, Germany) and centrifugation at 3,500 × g for 5 min.

Five mL of supernatant was pipetted into a 15 mL PP conical tube and 10 mg of sodium acetate was added to buffer the formic acid during the evaporation. The tube was then shaken for 5 min and centrifuged again for 1 min at 3500 × g at room temperature. To 1 mL of the extract, 10 µL of internal standard (IS) was added. Finally, 550 µL of supernatant was evaporated under nitrogen flow and 50 °C to dryness (Turbo Vap LV, Caliper Life Sciences, Hopkinton, MA, USA) and reconstituted with 500 µL injection solvent (ACN/Eluent A (25/75, v/v)). Finally, the sample was centrifuged again for 10 min at 17,530 × g (Microfuge R, Beckmann, Munich, Germany) and transferred into a Mini-UniPrep filter vial (Whatman, GE Healthcare, Little Chalfont, Buckinghamshire, UK) and was ready for subsequent LC–MS/MS and LC-HRMS analysis.

### Stock and working standard preparation

Stock solutions were used as certified reference solutions (AOH, AME, ^13^C_2_-TeA) or prepared from solids by weighing and dissolving in ACN (AME-d_3_) or ethanol (TeA, AOH-d_3_) before further dilution. For quantification, the standard mixture as well as the IS mixture were prepared in ACN/Eluent A (25/75, v/v). The mixture served both as a calibration mixture and as a spiking solution for validation.

### LC–MS/MS instrumentation and measurements

Analyses of wheat plants, nutrient solution and residual washing water were performed on a Shimadzu Nexera X2 HPLC system including binary pumps, a degasser, a column oven, an autosampler, and a control unit (Shimadzu Corporation, Duisburg, Germany) coupled to a QTrap 6500 + mass spectrometer (Sciex Germany GmbH, Darmstadt, Germany) equipped with an IonDrive™ Turbo V electrospray ionization (ESI) source. Chromatographic reversed-phase (RP) separation with 10 μL injection volume was achieved on a 100 × 2.0 mm, 3 μm particle size Gemini-NX C18 column with guard column (Phenomenex, Torrance, CA, USA) at a flow rate of 0.4 mL/min and a column oven temperature of 40 °C. The binary mobile phase consisted of water with 77 mg/L ammonium acetate, brought to pH 9 with ammonia (eluent A). Eluent B was composed of methanol/2-propanol (90/10, v/v). The gradient elution was performed as follows: 0–0.1 min B: 0–40%, 0.1–3.0 min B: 40–75%, 3.0–3.5 min B: 75–95%, 3.5–6.5 min B: 95%, 6.5–6.6 min B: 95–0%, 6.6–10 min B: 0%. MS detection was conducted using negative electrospray ionization (ESI) and measuring in multiple reaction monitoring mode (MRM) with mass transitions and MS conditions shown in Table S1. The following instrumental settings were applied: curtain gas 40, CAD medium, temperature 500 °C, ion spray voltage –4500 V, GS1 50, GS2 50. A diverter valve cut off the flow to the MS ion source before minute 0.8 and after minute 6.0. The MS/MS parameters used for quantification of AOH, AME and TeA are listed in Table S1.

### LC-HRMS/MS instrumentation and measurements

The samples were analyzed using an UltiMate 3000 UHPLC coupled to a Q Exactive Focus mass spectrometer (Thermo Fisher, Dreieich, Germany) equipped with a heated electrospray ionization (HESI-II) source. Chromatographic reversed-phase (RP) separation with 10 μL injection volume was achieved on a 100 × 2.1 mm, 1.6 μm particle size Cortecs UPLC C18 column with guard column (Waters, Milford, MA, USA) at a flow rate of 0.3 mL/min, and a column oven temperature of 40 °C. The binary mobile phase consisted of water (eluent A) and ACN (eluent B). The gradient elution was performed as follows: 0–2.0 min B: 0%, 2.0–10.0 min B: 0–40%, 10.0–15.0 min B: 40–80%, 15.0–16.0 min B: 80–100%, 16.0–18.0 B: 100%, 18.0–19.0 min B: 100–0%, 19.0–22.0 min B: 0%. High-resolution mass spectrometry (HRMS) was performed in negative ionization mode. The HESI-II temperature was set at 413 °C, the capillary temperature at 256 °C, the electrospray voltage at 3.8 kV, and S-Lens RF level at 60. Sheath and auxiliary gas flow rates were 37 and 7 L/min, respectively. All data in this study were acquired using a full scan mode covering the mass range from 80 to 1200 m*/z* with a resolution of 70,000 and automatic gain control (AGC) setting of 3 × 10^6^ with a maximum injection time (IT) of 100 ms. For confirmation, data-dependent MS2 (dd-MS^2^) was applied. In dd-MS^2^, the most abundant precursor ions in each full scan are selected by the quadrupole and then sent to the higher-energy collisional dissociation (HCD) cell for ion fragmentation and finally to the Orbitrap mass analyzer for detection. The dd-MS^2^ was performed at a mass resolution of 17,500, intensity threshold of 1.6e^5^, isolation width of 1.0 m*/z* and normalized collision energy (NCE) of 50% with ± 20% step.

**Fig. 2:**
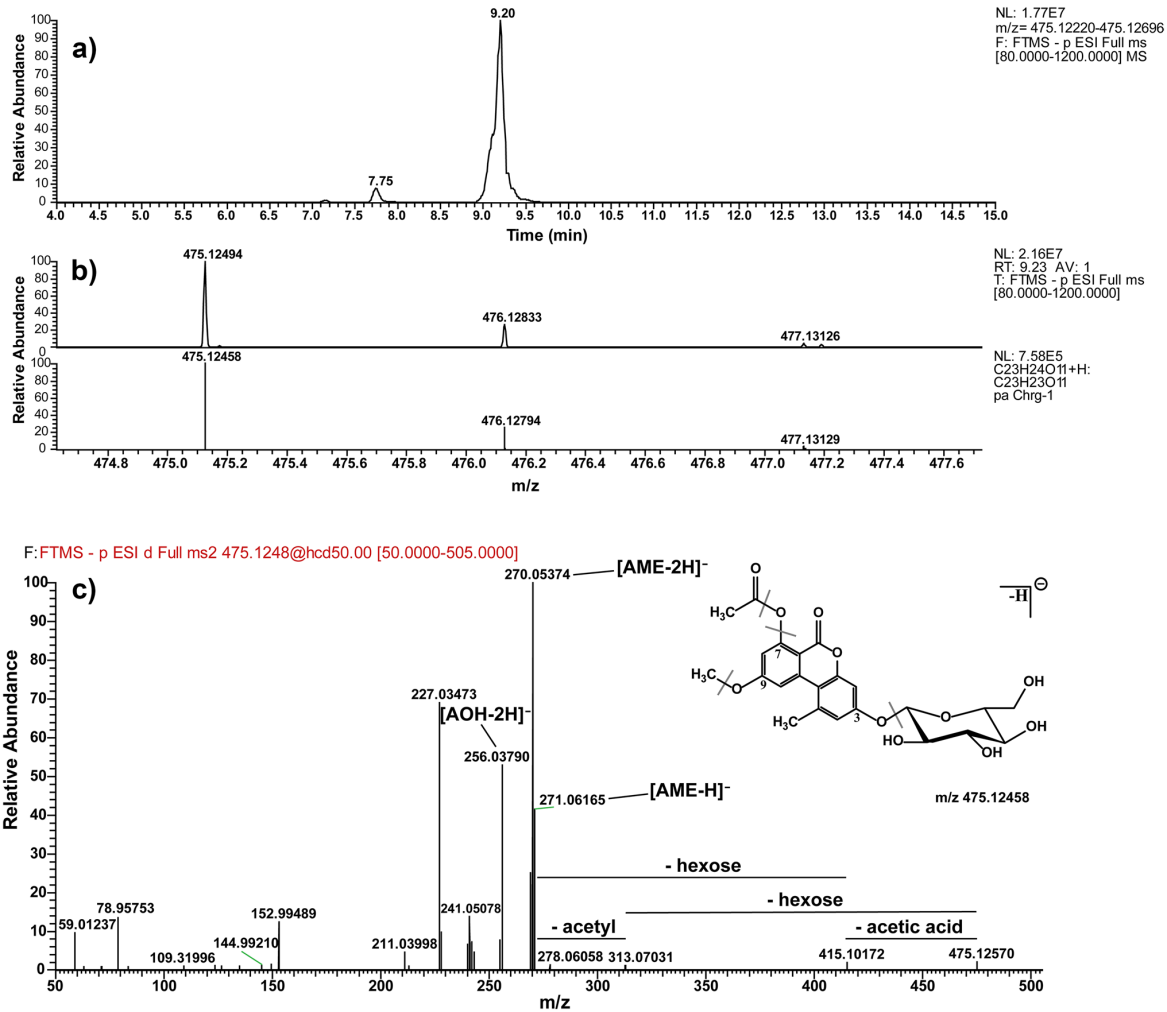
Conjugate identification steps using HRMS/MS. Application to AME-acetylhexoside. **a** Extracted ion chromatogram. **b** Comparison of the experimental and theoretical isotopic patterns. **c** MS^2^ spectrum and possible fragments. The structure shown is putative, and the actual positions may vary

The identification of conjugates was achieved through matching the accurate mass [M-H]^−^ and retention time with reference standards (whenever available). In the absence of reference standards, conjugates were proposed by their accurate masses [M-H]^−^, MS^2^ spectra, and/or isotopic patterns. The accurate mass and isotopic pattern of the measured conjugate should match the theoretical accurate mass and isotopic pattern of the proposed chemical formula within a mass tolerance of ± 5 ppm. The MS^2^ spectra should contain the quasimolecular ion of AOH and/or AME as product ions. When the abundance of a precursor ion is not sufficient to trigger fragmentation, the accurate mass and isotopic pattern criteria were solely used for identification. The conjugate identification steps are shown in Fig. 2.

### Method validation

The method was validated according to CEN/TS 16059 for wheat plant leaves and roots with a working range of 25–2500 µg/kg for TeA and 5–500 µg/kg for AOH and AME. The method performance parameters evaluated within the validation were as follows: linearity, working range, limit of detection (LOD), limit of quantification (LOQ), recovery, intermediate precision, and measurement uncertainty. Blank samples of 1 g wheat leaves and roots were weighed in 50 mL PP conical tubes, spiked with 60 μL of an *Alternaria* mycotoxin mix (AOH 60.0 µg/kg, AME 60.7 µg/kg, TeA 223 µg/kg), and left to dry in the dark at room temperature for 30 min before subsequent sample preparation. To determine the recovery rates and inter-day precision, three samples each of leaves and roots were spiked over 3 days resulting in 18 samples (final concentrations listed in Table 1). On the fourth day, samples of leaves and roots (10 each) were spiked at a low level for LOD and LOQ determination (AOH 5.0 µg/kg, AME 5.1 µg/kg, TeA 18.6 µg/kg). The four sample sets were measured in separate runs on different days by LC–MS/MS. Linearity was assessed using the German standard DIN 38402–51. LOD and LOQ were calculated according to the EURL Guidance Document on the Estimation of LOD and LOQ for Measurements in the Field of Contaminants in Feed and Food (Wenzl et al. 2016). The obtained validation values were satisfactory and are summarized in Table 1.

**Table 1:** Method validation parameters for leaves and roots. LOD and LOQ are estimated using spiked blank material

**Parameter**		**AOH**	**AME**	**TeA**
**Spiking concentration** **[µg/kg]**		60.0	60.7	223
**IS concentration in final** **calibration mix [ng/mL]**		21.4	22.7	97.7
**Calibration range** **[ng/mL]**		1.00–98.0	1.01–99.1	3.72–365
**Recovery ± U (*k* = 2) [%]**	**Leaves**	96 ± 11	91 ± 6	98 ± 16
	**Roots**	100 ± 16	101 ± 9	91 ± 13
**RSD interday [%]**	**Leaves**	5.7	3.2	8.0
	**Roots**	8.2	4.4	6.3
**LOD spiking concentration** **[µg/kg]**		5.00	5.06	18.6
**LOD [µg/kg]**	**Leaves**	4.8	2.3	8.4
	**Roots**	5.8	8.0	4.4
**LOQ [µg/kg]**	**Leaves**	16.0	7.5	27.7
	**Roots**	19.2	26.5	14.4
**Correlation coefficient (*r*)**	**Leaves**	0.999	0.999	0.994
	**Roots**	0.993	0.999	0.998

### Data analyses

Plant samples were extracted at least in duplicate and injected twice during LC–MS/MS measurements. LC–MS/MS data evaluation was performed with MultiQuant Software, ver. 3.0.2 (AB Sciex Germany GmbH, Darmstadt, Germany). LC-HRMS/MS data evaluation was performed with Xcalibur software, ver 4.4 (Thermo Fisher, Dreieich, Germany).

## Results

### Visual effects of mycotoxin treatment on wheat plant

The mycotoxin supplementation resulted in reduced growth of the plants after one and two weeks (Fig. S4). The wheat plants in the control group (both on the right) showed noticeably higher growth. In addition, the mycotoxin-treated plants exhibited yellowish discolored roots and more senescent leaves. The Hoagland nutrient solution was also remarkably cloudy with more suspended solids after 1 and 2 weeks, respectively, and exhibited a stronger odor compared to the control group. To examine the possibility of bacterial or fungal infections, samples of the nutrient solution were cultivated on nutrient media, but in no case microbial growth could be detected. Alternatively, the noticeable odor and turbidity in the mycotoxin-treated plant boxes could possibly be attributed either to physicochemical changes of ATs present at high concentrations or to the possibility that the roots formed odorous exudates in response to the treatment with ATs, but these hypotheses were not further investigated.

### Uptake of *Alternaria* toxins and quantification of TeA, AME, and AOH in the different plant compartments

**Fig. 3:**
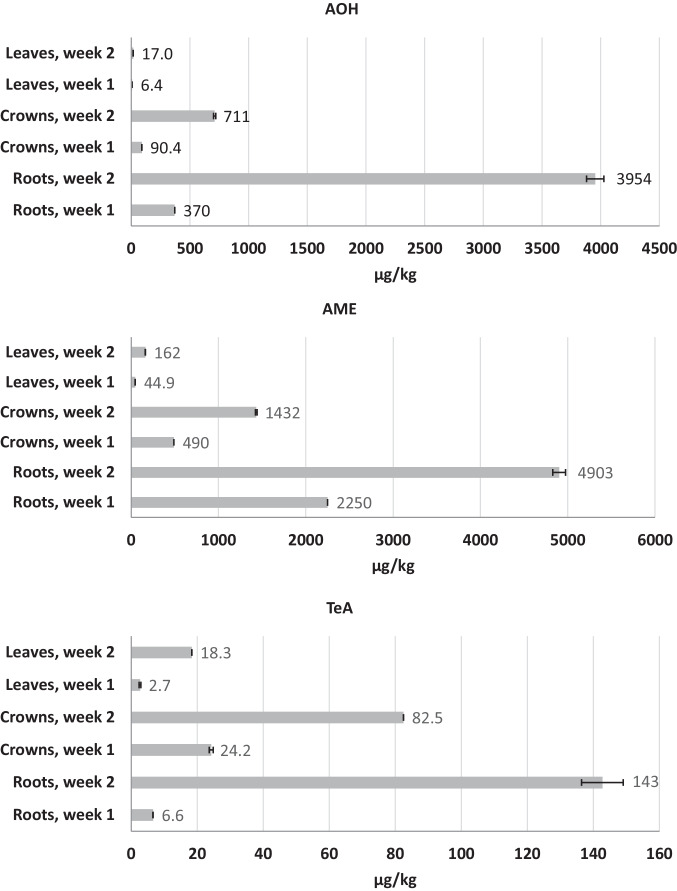
Concentration of AOH (top), AME (middle), and TeA (bottom) in the different plant parts after 1 and 2 weeks of cultivation expressed by the mean ± standard deviation

The quantification of ATs was performed in the three plant compartments; roots, crowns, and leaves (Fig. S2). In addition, the washing water of the roots and the remaining spiked Hoagland nutrient solution were analyzed. Figure 3 shows the determined levels of TeA, AME, and AOH in the plant compartments after 1 and after 2 weeks of cultivation. The corresponding control samples without AT supplementation showed no AOH and TeA content.

For all three ATs in all three plant compartments, an increase in the AT content can be seen over time when comparing the results after week one with that after week 2. Furthermore, the AT content seems to decrease along the transport away from the roots within the plant. Therefore, the highest contents of all three ATs were found in the roots (2 weeks after the application) and the lowest levels were found in the leaves (Fig. 3).

### Relative distribution of quantified ATs in the different plant compartments

**Fig. 4:**
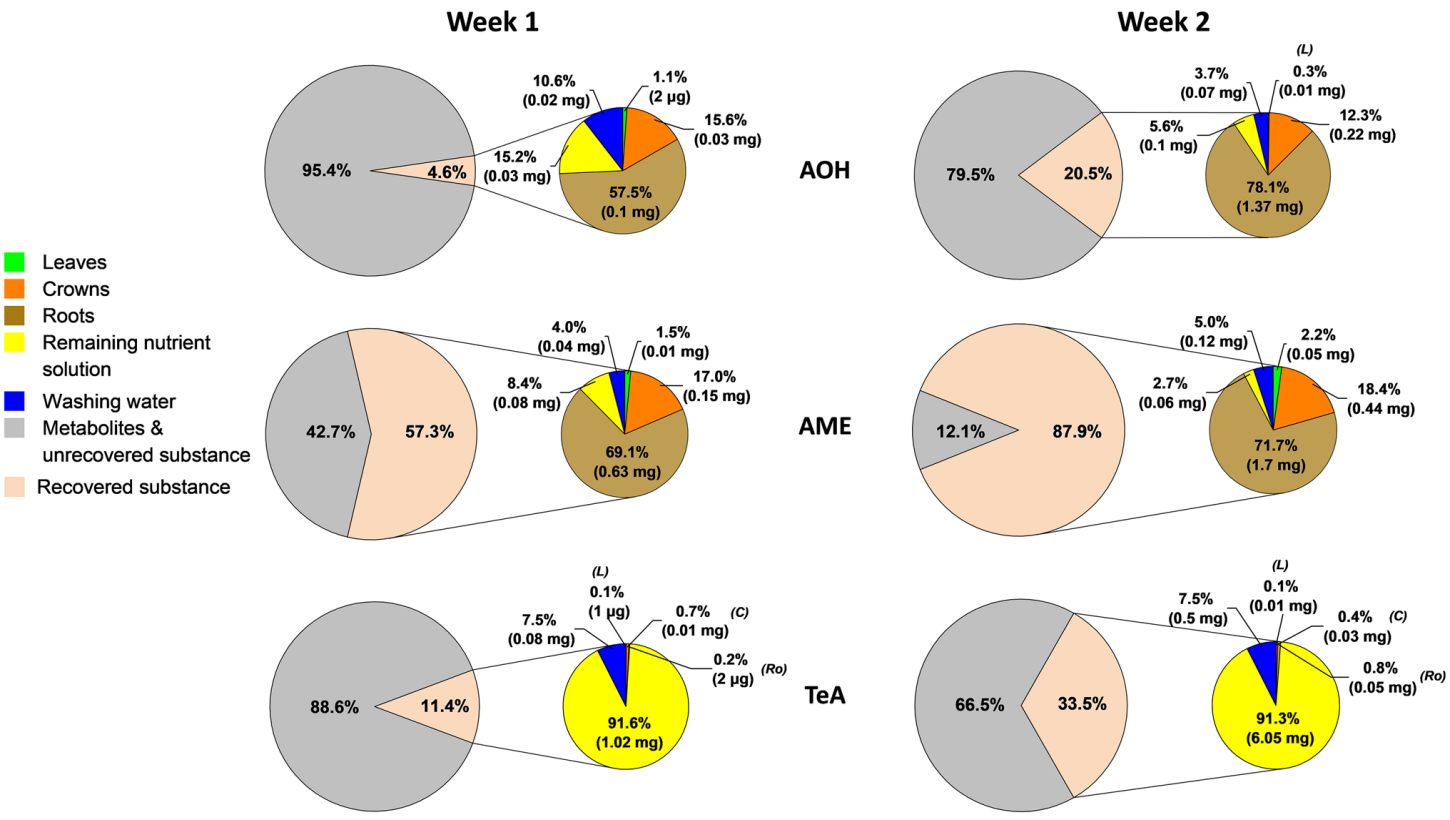
Distribution of AOH, AME, and TeA found (pink) in *Alternaria* toxin-treated roots (brown), crowns (orange), leaves (green), in the remaining nutrient solution (yellow), the washing water (blue), and the unknown (metabolites and unrecovered) remaining of the supplemented and quantified *Alternaria* toxins (grey) after 1 and 2 weeks of cultivation. Ro, roots; C, crowns; and L, leaves

The target AT concentrations in the Hoagland nutrient solution were 3.36 mg/L for AOH, 3 mg/L for AME, and 3.01 mg/L for TeA. As a control, aliquots of the freshly mixed solution (immediately after adding the mycotoxins to the Hoagland solution) were frozen and later analyzed, showing that the recovery for all three ATs was lower than the expected target concentration. The average actual concentrations determined were 1.0 mg/L for AOH, 0.4 mg/L for AME, and 2.5 mg/L for TeA. These actual values were used to calculate the relative distribution of the recovered and unrecovered fractions of ATs (Fig. 4, large pie chart). The recovered fractions were further broken down to show the relative distribution of ATs in the three analyzed plant compartments, the corresponding remaining nutrient solution and washing water after 1 and 2 weeks of cultivation (Fig. 4, small pie chart). In this context, it has to be emphasized that the following calculations consider only the three added ATs and not their metabolites which were not quantified.

For AME, around 57% of the added amount was recovered after 1 week of cultivation with AT-supplemented nutrient solution (Fig. 4, large pie chart). The detailed distribution of the recovered substance (Fig. 4, small pie chart) showed a heterogeneous distribution between the different compartments with the largest proportion detected in the roots with 69%, followed by 17% in the crowns and only around 2% in the leaves. After 2 weeks, the recovered fraction shifted to 88%, and the proportion found in roots slightly increased to 72%, in the crowns to 18% and in the leaves to 2%.

The distribution of the recovered AOH was similar to that of AME. After week 1, almost 58% of the recovered AOH was detected in the roots, followed by the crowns with 16% and only 1% in the leaves. However, the recovered fraction represented only 5% of the added AOH. After the second week, the recovered fraction increased to 21%. The distribution of the recovered fraction showed a noticeable increase in AOH content in the roots (78%). Although the percentage in the crowns and leaves has decreased, compared to week 1, to 12% and 0.3%, respectively, the absolute recovered amounts were more than in week 1. While AME and AOH behaved quite similarly in the plant, a different picture emerged for TeA. Here, a large part of the recovered TeA was found in the nutrient solution. After 1 week, the proportion was 92%, and after 2 weeks (supplementation with ATs on the 8th day) 91%. Likewise, a noticeable proportion was found in the washing water; 8% after 1 week and 8% after 2 weeks. Only very low proportions (less than 1% each) were found in the plant compartments compared to AOH and AME.

Concerning the proportion of the unrecovered AT fractions, it could be observed that the unrecovered AME fraction decreased from 43% after 1 week to 12% after 2 weeks. This percentage (12%) represented the lowest unrecovered fraction compared to AOH and TeA. AOH had the highest proportion of unrecovered whereabouts with 95% after 1 week and 80% after 2 weeks, followed by TeA with 89% after 1 week and 67% after 2 weeks. Thus, for all ATs, the percentage decreased remarkably after 2 weeks.

### Qualitative screening for AT conjugates

As revealed by the quantification of ATs in the different compartments and displayed in Fig. 4, a significant proportion of the added ATs were not recovered. Therefore, the plant samples were screened for AT conjugates formed by the wheat plants by metabolism of the ATs taken up from the nutrient solution. A list of previously reported AOH and AME conjugates have been screened using HRMS (Kohn 2019; Soukup et al. 2016). The AOH and AME conjugates that were detected and identified are shown in Fig. 1 and Tables 2 and 3. In contrast, no evidence for the formation of conjugates or other forms of metabolization of TeA was found. To the best of our knowledge, there are no known metabolites for TeA.

**Table 2:** Phase II metabolites of AOH annotated from roots, crowns and leaves analysis, and their qualitative abundance after 1 (W 1) and 2 (W 2) weeks of cultivation

**Peak number**	**RT (min)**	**Formula**	**Calculated *m/z *[M-H]**^**-**^	**Detected *m/z *[M-H]**^**-**^	**Mass error (ppm)**	**Identified/proposed compound**	**Occurrence**					
							**Roots**		**Crowns**		**Leaves**	
							**W 1**	**W 2**	**W 1**	**W 2**	**W 1**	**W 2**
*Compounds confirmed with reference standard by comparison of accurate mass, HR MS*^*2*^* spectra, and RT*												
**AOH-27**	10.64	C14H10O5	257.0456	257.0455	−0.18	**AOH**	+++ (1)	+++ (1)	+++ (1)	+++ (1)	++ (1)	++ (1)
**AOH-18**	7.67	C14H10O8S	337.0024	337.0029	1.51	**AOH-3-sulfate**	+++ (0.04)	++ (0.01)	++ (0.03)	+ (0.01)	N.D.	N.D.
**AOH-24**	8.20	C20H20O10	419.0984	419.0989	1.29	**9-*O*-*β*-D-glucopyranosyl-AOH (AOH-9-glucoside)**	++ (0.01)	+++ (0.05)	+ (0.01)	+ (0.01)	N.D.	N.D.
**AOH-26**	8.35	C20H20O10	419.0984	419.0988	1.07	**3-*O*-*β*-D-glucopyranosyl-AOH (AOH-3-glucoside)**	++ (0.01)	+++ (0.17)	+ (0.02)	++ (0.03)	+ (0.06)	+ (0.03)
**AOH-9**	7.01	C23H22O13	505.0988	505.0995	1.44	**9-*O*-(6′-*O*-malonyl-*β*-D-glucopyranosyl)AOH (AOH-6′-malonyl-9-glucoside)**	++ (0.02)	N.D.	+ (0.02)	N.D.	N.D.	N.D.
**AOH-14**	7.39	C23H22O13	505.0988	505.0992	0.82	**3-*O*-(6′-*O*-malonyl-*β*-D-glucopyranosyl)AOH (AOH-6′-malonyl-3-glucoside)**	N.D.	+++ (0.22)	N.D.	++ (0.01)	N.D.	N.D.
**AOH-16**	7.51	C26H30O15	581.1512	581.1523	1.97	**9-*O*-{*β*-D-glucopyranosyl(1→6)-*β*-D-glucopyranosyl}AOH (AOH-9-diglucoside)**	+ (0.01)	+ (0.01)	N.D.	N.D.	N.D.	N.D.
*Proposed conjugates*												
**AOH-11**	7.06	C14H10O8S	337.0024	337.0030	1.87	**AOH-7/9-sulfate**^**a**^	+ (0.01)	++ (0.01)	+ (0.01)	+ (0.01)	N.D.	N.D.
**AOH-1**	4.98	C14H10O11S2	416.9592 (z=2: 207.9760)	207.9758 (z=2)	−1.83	**AOH-3,9-disulfate**^**b**^	+ (0.01)	+ (0.01)	+ (0.01)	+ (0.01)	N.D.	N.D.
**AOH-10**	7.02	C22H22O11	461.1089	461.1096	1.49	**AOH-acetylhexoside**^**a**^	+++ (0.06)	+++ (0.68)	++ (0.09)	N.D.	N.D.	N.D.
**AOH-13**	7.37	C22H22O11	461.1089	461.1095	1.29	**AOH-acetylhexoside**^**a**^	N.D.	+++ (0.57)	N.D.	+++ (0.06)	N.D.	N.D.
**AOH-8**	6.76	C20H20O13S	499.0552	499.0560	1.63	**AOH-3/9-sulfate-9/3-hexoside**^**a**^	+ (0.01)	++ (0.02)	+ (0.01)	+ (0.01)	N.D.	N.D.
**AOH-3**	5.77	C25H28O14	551.1406	551.1417	1.87	**AOH-pentosylhexoside**^**b**^	+ (0.01	+ (0.01+	+ (0.02)	++ (0.01)	N.D.	N.D.
**AOH-20**	7.98	C25H28O14	551.1406	551.1415	1.62	**AOH-pentosylhexoside**^**a**^	+ (0.01)	++ (0.01)	N.D.	N.D.	N.D.	N.D.
**AOH-15**	7.43	C26H30O14	565.1563	565.1572	1.61	**AOH-desoxyhexosylhexoside**^**b**^	+ (0.01)	N.D.	+ (0.02)	+ (0.01)	++ (1.08)	++ (0.55)
**AOH-19**	7.91	C26H30O14	565.1563	565.1574	1.90	**AOH-desoxyhexosylhexoside**^**a**^	+ (0.01)	++ (0.01)	N.D.	N.D.	N.D.	N.D.
**AOH-22**	8.11	C26H30O14	565.1563	565.1569	1.03	**AOH-desoxyhexosylhexoside**^**a**^	+ (0.01)	++ (0.01)	N.D.	N.D.	N.D.	N.D.
**AOH-25**	8.25	C26H30O14	565.1563	565.1573	1.84	**AOH-desoxyhexosylhexoside**^**a**^	+ (0.01)	++ (0.01)	N.D.	N.D.	N.D.	N.D.
**AOH-6**	6.24	C26H30O15	581.1512	581.1522	1.72	**AOH-dihexoside**^**b**^	+ (0.01)	N.D.	+ (0.01)	+ (0.01)	+ (0.5)	+ (0.12)
**AOH-17**	7.63	C26H30O15	581.1512	581.1522	1.66	**AOH-Dihexoside**^**a**^	+ (0.01)	++ (0.01)	+ (0.01)	N.D.	N.D.	N.D.
**AOH-21**	8.07	C26H30O15	581.1512	581.1519	1.25	**AOH-dihexoside**^**b**^	+ (0.01)	+ (0.01)	N.D.	N.D.	N.D.	N.D.
**AOH-23**	8.19	C26H30O15	581.1512	581.1521	1.56	**AOH-dihexoside**^**a**^	+ (0.01)	++ (0.01)	+ (0.01)	+ (0.01)	+ (0.04)	N.D.
**AOH-2**	5.06	C29H32O18	667.1516	667.1522	0.90	**AOH-malonyldihexoside**^**b**^	N.D.	+ (0.01)	N.D.	N.D.	N.D.	N.D.
**AOH-5**	5.85	C29H32O18	667.1516	667.1512	−0.61	**AOH-malonyldihexoside**^**a**^	+ (0.01)	++ (0.01)	N.D.	N.D.	N.D.	N.D.
**AOH-7**	6.75	C29H32O18	667.1516	667.1522	0.86	**AOH-malonyldihexoside**^**b**^	+ (0.01)	N.D.	N.D.	0.01	0.51	0.15
**AOH-12**	7.19	C29H32O18	667.1516	667.1526	1.50	**AOH-malonyldihexoside**^**a**^	N.D.	++ (0.01)	N.D.	N.D.	N.D.	N.D.
**AOH-4**	5.80	C32H34O21	753.1520	753.1538	2.47	**AOH-dimalonyldihexoside **^**b**^	N.D.	N.D.	+ (0.01)	+ (0.01)	+ (0.03)	+ (0.02)

*N.D.* not detected, + detected with low relative abundance (intensity <1.2e6), ++ detected with medium relative abundance (1.2e6< intensity <1.2e7), +++ detected with high relative abundance (intensity >1.2e7). The numbers in parentheses are the ratio of intensity of conjugate to that of AOH in the respective sample

^a^Annotation with accurate mass, isotopic pattern and HR MS^2^ spectra

^b^Annotation with accurate mass and isotopic pattern

**Table 3:** Phase II metabolites of AME annotated from roots, crowns and leaves analysis and their qualitative abundance after one (W 1) and 2 (W 2) weeks of cultivation

**Peak number**	**RT (min)**	**Formula**	**Calculated *m/z* [M-H]**^**−**^	**Detected *m/z* [M-H]**^**−**^	**Mass error (ppm)**	**Identified/proposed compound**	**Occurrence**					
							**Roots**		**Crowns**		**Leaves**	
							**W 1**	**W 2**	**W 1**	**W 2**	**W 1**	**W 2**
*Compounds confirmed with reference standard by comparison of accurate mass, HR MS*^*2*^* spectra, and RT*												
**AME-19**	12.87	C15H12O5	271.0612	271.0614	0.64	**AME**	+++ (1)	+ ++ (1)	+++ (1)	+++ (1)	++ (1)	+++ (1)
**AME-16**	9.50	C15H12O8S	351.0180	351.0183	0.94	**AME-3-sulfate**	+++ (0.33)	+++ (0.03)	++ (0.13)	++ (0.06)	N.D.	N.D.
**AME-8**	8.70	C21H22O10	433.1140	433.1148	1.75	**7-*O*-*β*-D-glucopyranosyl-AME (AME-7-glucoside)**	+ (0.01)	++ (0.01)	+ (0.01)	+ (0.01)	++ (0.38)	++ (0.02)
**AME-17**	10.13	C21H22O10	433.1140	433.1146	1.34	**3-*O*-*β*-D-glucopyranosyl-AME (AME-3-glucoside)**	++ (0.01)	++ (0.01)	+ (0.01)	+ (0.01)	N.D.	+ (0.01)
**AME-15**	9.40	C24H24O13	519.1144	519.1151	1.23	**3-*O*-(6′-*O*-malonyl-*β*-D-glucopyranosyl) AME (AME-6‘-malonyl-3-glucoside)**	N.D.	N.D.	N.D.	N.D.	+ (0.16)	+ (0.01)
*Proposed conjugates*												
**AME-7**	7.74	C23H24O11	475.1246	475.1253	1.59	**AME-acetylhexoside**^**a**^	+ (0.01)	++ (0.01)	+ (0.01)	++ (0.02)	++ (0.31)	++ (0.02)
**AME-13**	9.19	C23H24O11	475.1246	475.1249	0.68	**AME-acetylhexoside**^**a**^	++ (0.01)	+++ (0.05)	+ (0.01)	++ (0.03)	N.D.	+ (0.01)
**AME-18**	10.57	C23H24O11	475.1246	475.1249	0.71	**AME-acetylhexoside**^**b**^	N.D.	N.D.	+ (0.01)	++ (0.01)	+ (0.11)	++ (0.02)
**AME-8**	8.70	C21H22O13S	513.0708	513.0715	1.24	**AME-3/7-sulfate-7/3-hexoside**^**a**^	+ (0.01)	++ (0.01)	+ (0.01)	+ (0.01)	N.D.	+ (0.01)
**AME-14**	9.25	C24H24O13	519.1144	519.1151	1.34	**AME-malonylhexoside (**possibly **AME-6′-malonyl-7-glucoside)**^**a**^	N.D.	++ (0.01)	N.D.	+ (0.01)	+ (0.06)	+ (0.01)
**AME-10**	8.86	C26H30O14	565.1563	565.1569	1.13	**AME-pentosylhexoside**^**b**^	N.D	N.D	+ (0.01)	+ (0.01)	++ (0.24)	++ (0.02)
**AME-2**	6.23	C27H32O15	595.1668	595.1679	1.69	**AME-dihexoside**^**b**^	++ (0.01)	++ (0.01)	++ (0.02)	+ (0.01)	+ (0.04)	+ (0.01)
**AME-4**	6.47	C27H32O15	595.1668	595.1680	1.89	**AME-dihexoside**^**b**^	+ (0.01)	++ (0.01)	N.D.	+ (0.01)	+ (0.20)	+ (0.01)
**AME-6**	7.68	C30H34O18	681.1672	681.1689	2.47	**AME-malonyldihexoside**^**b**^	N.D.	N.D.	N.D.	N.D.	+ (0.19)	+ (0.01)
**AME-11**	8.92	C30H34O18	681.1672	681.1686	1.97	**AME-malonyldihexoside**^**a**^	+ (0.01)	++ (0.01)	N.D.	+ (0.01)	N.D.	N.D.
**AME-12**	9.05	C30H34O18	681.1672	681.1683	1.52	**AME-malonyldihexoside**^**b**^	N.D.	++ (0.01)	N.D.	+ (0.01)	N.D.	N.D.
**AME-1**	6.14	C33H42O20	757.2197	757.2218	2.82	**AME-trihexoside**^**b**^	+ (0.01)	+ (0.01)	+ (0.01)	+ (0.01)	N.D.	N.D.
**AME-3**	6.32	C33H42O20	757.2197	757.2214	2.34	**AME-trihexoside**^**b**^	N.D.	+ (0.01)	N.D.	+ (0.01)	+ (0.03)	N.D.
**AME-5**	6.61	C33H36O21	767.1676	767.1703	3.47	**AME-dimalonyldihexoside**^**b**^	N.D.	N.D.	+ (0.01)	+ (0.01)	N.D.	N.D.

*N.D.* not detected, + detected with low relative abundance (intensity <1.2e6), ++ detected with medium relative abundance (1.2e6< intensity <1.2e7), +++ detected with high relative abundance (intensity >1.2e7). The numbers in parentheses are the ratio of intensity of conjugate to that of AME in the respective sample

^a^Annotation with accurate mass, isotopic pattern and HR MS^2^ spectra

^b^Annotation with accurate mass and isotopic pattern

### Structure elucidation of the conjugates using the example of AME acetylhexoside

The identification procedure is exemplarily presented for AME acetylhexoside in Fig. 2, showing the corresponding extracted ion chromatogram (Fig. 2a) from a root sample. Subsequently, the experimental isotope pattern was visually compared with the theoretical one (Fig. 2b) to confirm the molecular formula of the proposed conjugate. In the final step of the identification workflow, the parent ion was fragmented and the fragments were analyzed for typical mass losses (Fig. 2c). In the case of the AME acetylhexoside, these were the loss of the hexose and of the acetyl groups to yield the *m/z* of AME that was further fragmented to AOH. The assignment of the exact substituent position is not possible via this workflow; for this purpose, reference standards should be used whenever possible.

### Conjugates of AOH in roots, crowns, and leaves of wheat plants

The screening was performed for typical phase I metabolites such as hydroxylated or dehydrogenated forms, but no phase I metabolites were detected. However, as can be seen in Table 2, a variety of possible phase II metabolites was detected. Among the 26 detected AOH conjugates, the identity of six conjugates was confirmed by comparison with reference standards. In Fig. S5, the overlaid extracted ion chromatograms of the AOH conjugates found in the roots (after 2 weeks of application) are shown exemplarily. Similar to the parent compound AOH, its metabolites were most abundant in the roots and least abundant in the leaves. In many cases, the intensity of the conjugate signals increased in the samples taken at the end of week 2. Especially the conjugates with sugar molecules were detected most frequently, particularly in the roots. The AOH-3 and 9-glucoside, AOH-acetylhexosides, the two malonyl-glucoside forms, and also the AOH-3-sulfate were found mainly in the roots. In roots supplemented for 2 weeks, the highest intensities were found for AOH-acetylhexosides, followed by AOH-glucosides and AOH-6′-malonyl-3-glucoside.

Phase II metabolites were also detected in the crowns, but in smaller amounts compared to the roots. Here, the predominant conjugates were AOH-3-sulfate, AOH-3-glucoside, AOH-acetylhexosides, AOH-6′-malonyl-9-glucoside, and AOH-pentosylhexoside. Again, AOH-acetylhexosides were the most abundant metabolites in the crowns. Specifically for AOH, the AOH-disulfate has been detected in the roots and crowns. In the leaves, the least number of metabolites overall was detected, which showed also less intensive signals and include AOH-3-glucoside, AOH-desoxyhexosylhexoside, and the AOH-dihexoside. Furthermore, AOH-malonyldihexoside was detected as well as AOH-dimalonyldihexoside. The most abundant metabolites in the leaves were AOH-desoxyhexosylhexoside, AOH-dihexoside, and AOH-malonyldihexoside. The intensity of the AOH-desoxyhexosylhexoside signal in the leaves samples of week 1 was even higher than the AOH signal itself.

### Conjugates of AME in roots, crowns and leaves of wheat plants

Figure S6 shows an example of the overlaid extracted ion chromatograms of the AME conjugates found in root samples after 2 weeks. A total of 18 AME conjugates were detected and identified in the three plant compartments, four of which by comparison with reference standards (Table 3). As described for AOH, no typical phase I metabolites, apart from AOH, were detected. AOH is a possible metabolite of AME, and if it would have been formed, it would not be possible to identify it as AOH was also part of the spiking solution. Analogous to AOH, the AME signal was detected in high intensity in all plant compartments after 1 and 2 weeks, and no AME was detected in the control samples. The most abundant metabolite was AME-3-sulfate which was found mainly in the roots with the most intense signal and also in the crowns after 1 and 2 weeks. However, no AME-3-sulfate was detected in the leaves. The majority of AME metabolites in the leaves was formed by AME-7-glucoside, AME-acetylhexosides, AME-6′-malonyl-3-glucoside, AME-pentosylhexoside, AME-dihexoside, and AME-malonyldihexoside. AME-7 glucoside, AME-acetylhexoside, and a dihexoside form were found in all three plant compartments. Conjugates with sugar molecules were also predominant for AME. The distribution of conjugates in plant compartments was more heterogeneous compared to AOH, where the majority was found in the roots. Furthermore, an AME hexoside sulfate was found, but due to the lack of reference standards, the assignment of the localization of the conjugation within the molecule was not possible. A similar form had also been found for AOH. No AME-disulfate has been detected in any plant compartment. In contrast to AOH, the formation of two AME trihexosides in the roots, crowns, and leaves could be detected for AME. For further information, the extracted ion chromatograms and respective MS and MS^2^ spectra for all detected AOH and AME conjugates can be found in the supplementary information (Figs. S7–S52). In addition, no conjugates could be detected in the control plants nor the nutrient solution at any time point.

## Discussion

### *Alternaria* toxins and their phytotoxic activities

The phytotoxic activity of AOH, AME and TeA has received little attention so far. The cyto- and genotoxicity of AOH and AME have been studied rather than their phytotoxic properties. As Wang et al. reviewed, AOH possessed a cytotoxic activity in soybean cell cultures, and AME inhibited the electron transport system in spinach chloroplasts (Wang et al. 2022). Hildebrand et al. reported that AOH and AME induced a reduction of cell mass and an increase of percentage of dead cells in a tobacco cell culture after 72 h, but to a different extent for AOH and AME (Hildebrand et al. 2015). Overall, few studies on the phytotoxic activity of dibenzopyranones exist; therefore, it is difficult to draw comparisons to the observations of the present experiment. The visually recognizable smaller wheat plants in the AT-supplemented nutrient solutions may well be due to a combined phytotoxic activity of the three ATs, but a proof is still lacking. A hint would come from the investigations of Tang et al., who observed that AOH inhibited root growth of *Pennisetum alopecuroides*, *Medicago sativa*, and *Amaranthus retroflexus* at a concentration of 1000 µg/mL (Tang et al. 2020). In general, the impact of the phytotoxic activities of ATs and their conjugates is an important factor that needs to be investigated in future studies.

### Stability of *Alternaria* toxins

It has been found that the recovery for all three ATs in Hoagland nutrient solution was lower than the expected target concentration. Although there is no clear explanation for this reduction in concentration, it could be assumed to be due to degradation, adsorption, and/or precipitation of ATs. The stability of AOH, AME, and TeA in different solvents under controlled conditions has been tested over 3 months and showed a continuous decrease in AOH and AME while the concentration of TeA remained relatively unchanged (data not shown). In our study, the cultivation conditions (light, oxygen, metals in nutrient solution, etc.) may accelerate the degradation of ATs (Bazin et al. 2013; Keller et al. 2017; Kotthoff et al. 2019). However, these factors were not systematically investigated in our study.

### Uptake of AOH, AME, and TeA in the wheat plant compartments

This is the first study demonstrating the uptake of ATs in vivo using a hydroponic system and whole wheat plants examining both the distribution of ATs within the plant and the modification of ATs by the wheat plants. This distribution was in a time-dependent fashion where the concentrations of ATs in samples collected after 2 weeks of cultivation were higher than those after 1 week in the respective plant compartments. Therefore, the potential accumulation of ATs in the grains cannot be excluded. The incorporation of ATs in grains is not investigated in this study because the hydroponic system is unsuitable for growing to mature grains especially with the high AT concentrations employed in the study.

It was shown that among the three plant compartments, the root samples had the highest concentrations of the added ATs after both 1 and 2 weeks. With both AOH and AME having limited solubility in water, they could be adsorbed to the surface of the roots preventing them from entering the plant even after using sterile aqueous solution for washing and thus contributing to the proportions detected in the roots. However, the contribution of this proposed effect could be considered to be minimal: on the one hand, low concentrations of AOH and AME were detected in the washing solution (the concentrations of AOH and AME in the water after second washing step were about 30–50% less than that after first washing). On the other hand, only the presence of ATs inside the roots can explain the transport of ATs and their metabolites into the leaves.

Concentrations of AOH, AME, and TeA were higher in roots than in crowns or leaves. TeA was generally the least abundant among the 3 ATs in all plant compartments, although the added concentration in the nutrient solution was even higher for TeA compared to AOH and AME.

Malchi et al. demonstrated in a field study the uptake of different ionic and non-ionic pharmaceutical compounds by root vegetables following irrigation with treated wastewater. The authors showed that non-ionic compounds were detected in crops at significantly higher concentrations than ionic ones (Malchi et al. 2014). The non-ionic organic compounds are known to easily cross the cell membranes and therefore have a higher potential to be taken up by roots and then being translocated and therefore accumulate to a higher proportion in the leaves (Briggs et al. 1982). However, the much lower permeability of cell membranes to ionic compounds as well as the possible adsorption to the soil and the cell wall could explain the relatively lower concentration of ionic compounds detected in crops. In addition, weak acids with pKa values below the pH in the soil will be negatively charged in the soil and therefore get repulsed by the negative charge of the cells of the root apoplast hindering the uptake (Trapp 2004). This could explain the reduced uptake of TeA (pKa 3.5) by the roots from the nutrient solution (pH ~ 5.7) in our study. Since TeA preferentially forms complexes with the metal cations of the salts present in the nutrient solution such as copper or zinc, which are more stable and highly soluble in water, this possibly resulted in reduced uptake by roots.

The uptake of mycotoxins in this hydroponic trial can arguably take place far more intensively than in a trial under field conditions. In the latter, it must be assumed that a considerable proportion of the ATs would be bound to soil particles or contained in complex substances such as humic acids, and uptake into the plant would thus be reduced. However, other studies have shown that mycotoxins such as AFB1 are also taken up and stored by the plant under field conditions (Hariprasad et al. 2013). This has also been described for other substance classes such as active compounds in hygiene products or pharmaceutical substances (Wu et al. 2013) and veterinary antibiotics (Grote et al. 2007; Schwake-Anduschus and Langenkämper 2018).

Further investigation of the uptake of ATs by plants should include field studies to examine the interaction between ATs and soil particles, focusing on the uptake of different ATs by plants under environmental conditions and on the interaction between ATs and soil particles. Studies with naturally contaminated soils and experiments with lower concentrations that reflect the situation in the naturally contaminated soil are needed. In addition, field studies should also investigate the incorporation of ATs in the edible parts (grains). The data obtained from such studies could help elucidate the different sources of contamination and could serve as a basis for a differentiated exposure estimate in the context of risk assessment.

### Modification of AOH and AME by wheat plants

Due to their sessile lifestyle, plants have a distinct enzyme system to metabolize potentially harmful xenobiotics to detoxify them. These enzymes include the O-malonyltransferases, sulfotransferases, and O-glucosyltransferases, which are responsible for the formation of conjugates in phase II metabolism. The numerous AOH and AME conjugates which have been detected in this study reflect this diversity of enzyme reactions. The free phenolic hydroxyl groups in AOH and AME also allow the formation of mixed conjugates such as sulfoglucoside and acetylglucosides. It can be assumed that there are even more conjugates which, however, cannot be fully detected following our extraction procedure. O-malonyl-glucoside conjugates for example are difficult to isolate, and, therefore, it could be possible that the glucosides were previously present as malonyl glucosides and the malonyl moiety was cleaved during extraction (Sandermann 1999). In addition, during processing, the cleavage of malonyl-glucosides to acetyl-glucosides has been reported for flavonoids (Coward et al. 1998; Lee et al. 2022) which cannot be excluded by our data. A systematic multi-target or non-target screening could also reveal other metabolites but that was not within the scope of this work. Although the metabolites detected in this study could in part explain the gap between the supplemented and recovered amounts of AOH and AME, there is still a considerable unrecovered fraction that could be attributed to the presence of other metabolites, missed conjugates due to suboptimal MS source parameters (e.g., glutathione and cysteine), covalent binding of conjugates to cell walls, and/or the potential degradation, adsorption, or precipitation of ATs under the cultivation conditions (Berthiller et al. 2013; Coleman et al. 1997; Zhou et al. 2018).

In this study, the conjugates with sugar molecules were detected most frequently, particularly in the roots. Among hexosyl conjugates, AOH-acetylhexosides were the most abundant AOH metabolites in the roots and crowns. Conjugates that contain more than one sugar moiety form the majority of conjugates found in leaves. Considerable amounts of AME-7-glucoside, AME-acetylhexosides, AME-6′-malonyl-3-glucoside were also detected in leaves. On the other hand, AME-3-sulfate formed the bulk of the detected AME metabolites in roots and crowns.

One of the most important plant mechanisms for detoxification is conjugation with glucose as described for example for DON in durum wheat by Righetti et al. (Righetti et al. 2020). Studies, albeit few, have also been published for AOH and AME showing that conjugation can occur in plant cells and fungi. Hildebrand et al. showed that both ATs are conjugated to a large extent in suspension cultures of BY-2 tobacco cells. *β*-D-glucopyranosides were identified (bound in the 3- or 9-position of AOH) as well as their 6′-malonyl derivatives and a gentiobiose conjugate (Hildebrand et al. 2015). For AME, conjugation led to the D-glucopyranoside (mostly bound in the 3-position of AME) and its 6′- and 4′-malonyl derivatives. These conjugates could now also be detected in wheat plants in the various plant parts in this study, showing that the wheat plant as a whole also metabolizes uptaken AOH and AME to various conjugates in vivo. Sulfate conjugates (monosulfate, disulfate, and sulfohexoside) were also formed in wheat plants after 1 week and 2 weeks. For ZEN, the formation of sulfate conjugates besides the more common glucose and malonyl conjugates was described in wheat plants, but to a lesser extent (Righetti et al. 2017). The authors described reductive and oxidative hydroxylation, followed by glycosylation and malonyl conjugation, as the major biotransformation pathways of ZEN as detoxification mechanism of the wheat plant, even in the absence of *Fusarium* infection.

In this study, it could be clearly demonstrated that a significant proportion of the supplemented AOH and AME was taken up by the wheat plants from the nutrient solution via the roots and distributed within the plant up to the leaves. In addition, numerous conjugates have been detected and identified. It can be assumed that some of the *Alternaria* toxins are metabolized in the course of detoxification and that the conjugates are permanently stored in the plant compartments with the possibility to enter the food chain via the ripening ears. To which extent this entry pathway adds to the overall exposure to these toxins remains to be further investigated. Furthermore, the potential transfer of conjugates from plant debris into the soil and subsequent absorption by plants needs also to be studied. It will certainly be interesting in future studies to cultivate the plants until the ripening of the wheat ears and then investigate the amounts of ATs and their metabolization within the kernels. The data shown in this study could represent an important first step for a differentiated exposure estimation in the context of risk assessment.

## Supplementary Information

Below is the link to the electronic supplementary material.

Supplementary file1 (DOCX 9377 KB)
