# Supplementary material for: Root uptake and metabolization of Alternaria toxins by winter wheat plants using a hydroponic system
Source: Mycotoxin Res. 2023 Mar 16;39(2):109–26. doi: 10.1007/s12550-023-00477-3 (PMC10181980; doi:10.1007/s12550-023-00477-3)
Supplement: Supplementary file 1 — Supplementary file1 (DOCX 9377 KB) [file 12550_2023_477_MOESM1_ESM.docx]

**Root uptake and metabolization of *Alternaria* toxins by winter wheat plants using a hydroponic system**

Julia Jaster-Keller^1^, Marina E.H. Müller^2^, Ahmed H. El-Khatib^1,*^, Nicole Lorenz^1^, Arnold Bahlmann^1^, Ulrike Mülow-Stollin^1,^^[[1]](#footnote-1)^§, Mirko Bunzel^3^, Sophie Scheibenzuber^4^, Michael Rychlik^4^, Grit von der Waydbrink^2^, Stefan Weigel^1^

^1^German Federal Institute for Risk Assessment (BfR), Department for Safety in the Food Chain, Max‑Dohrn‑Str. 8‑10, 10589 Berlin, Germany

^2^Leibniz Centre for Agricultural Landscape Research (ZALF), Eberswalder Str. 84, 15374 Müncheberg, Germany

^3^Karlsruhe Institute of Technology (KIT), Institute of Applied Biosciences, Department of Food Chemistry and Phytochemistry, South Campus, Adenauerring 20 A, Karlsruhe, Germany

^4^Technical University of Munich (TUM), Chair of Analytical Food Chemistry, Department of Life Science Engineering, Maximus-von-Imhof Forum 2, 85354 Freising, Germany

* Corresponding author: ahmed.el-khatib@bfr.bund.de

**Table S1.** Mass transitions and conditions for LC-MS/MS quantification.

| **Analyte** | **[M-H]**^-^ ***(m/z*)** | **Product ions Q/q (*m/z*)** | **Collision energy**  **(CE) [eV]** | **Collision cell exit potential (CXP) [V]** | **Declustering potential**  **(DP) [V]** | **Expected RT (min)** |
| --- | --- | --- | --- | --- | --- | --- |
| **AOH** | 257 | 215/147 | -33/-46 | -11/-9 | -80 | 2.9 |
| **AOH-d_3_** | 260 | 218 | -36 | -11 | -80 | 2.9 |
| **AME** | 271 | 256.1/228 | -30/-40 | -11/-17 | -75 | 4.9 |
| **AME-d_3_** | 274 | 259.1 | -30 | -11 | -75 | 4.9 |
| **TeA** | 196.1 | 139/112 | -26/-32 | -9/-7 | -50 | 2.3 |
| **^13^C_2_-TEA** | 198.1 | 141 | -26 | -9 | -50 | 2.3 |


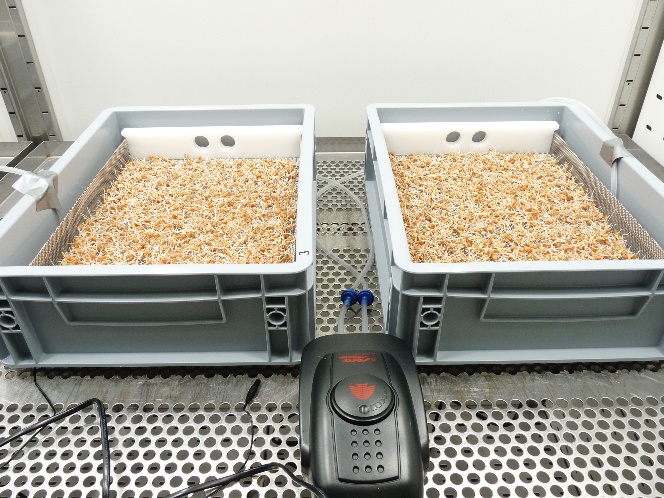


**Fig. S1.** The germination boxes with the germinated winter wheat seeds on the perforated insert of the boxes.


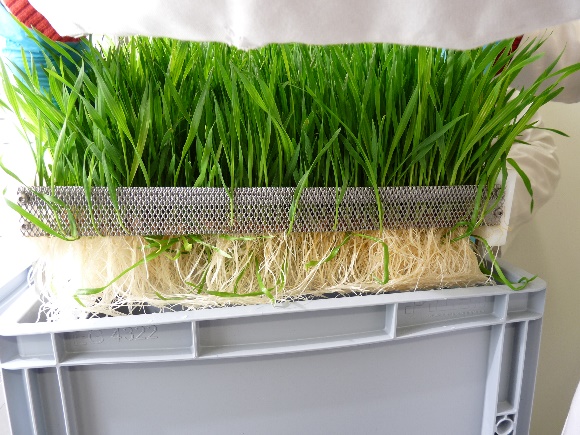

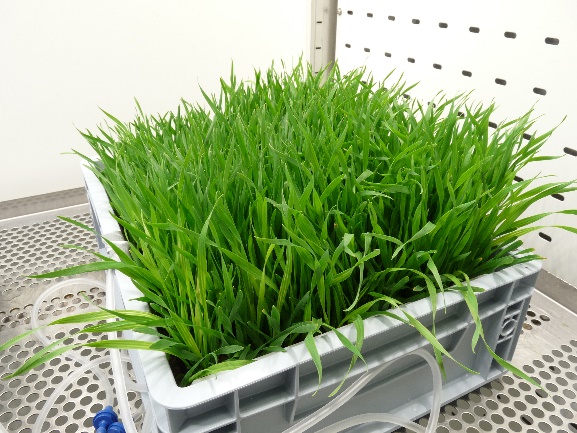


**Leaves**

**Crowns**

**Rootss**

**Fig. S2.** Winter wheat plants (cultivar “Julius”) after two weeks in germination boxes growing in Hoagland nutrient solution. At this development stage, the mycotoxin solution (consisting of AOH, AME and TeA) was added to the nutrient solution.


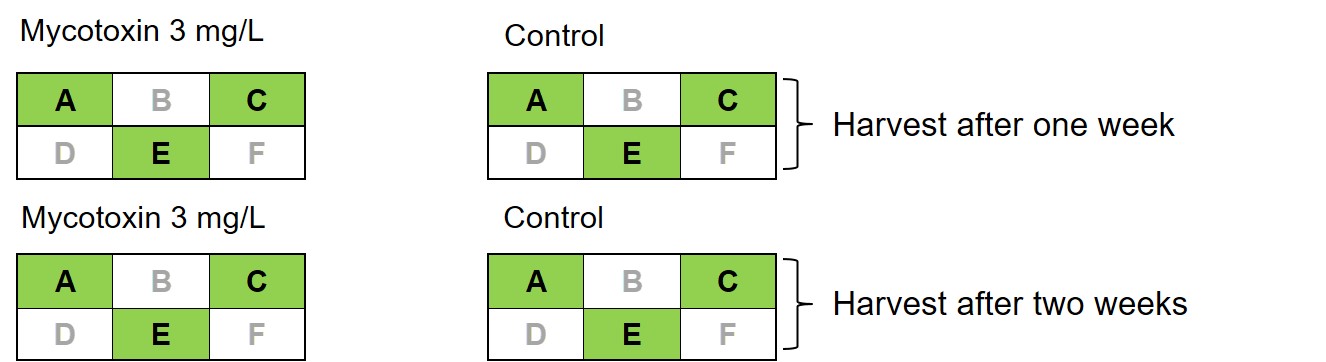


**Fig. S3.** Experimental design with subdivision of plants in the control group and the mycotoxin test group, pooling of areas A, C, E. In the mycotoxin test group, the concentration of each of the mycotoxins (AOH, AME, TeA) in the germination box was 3 mg/L.


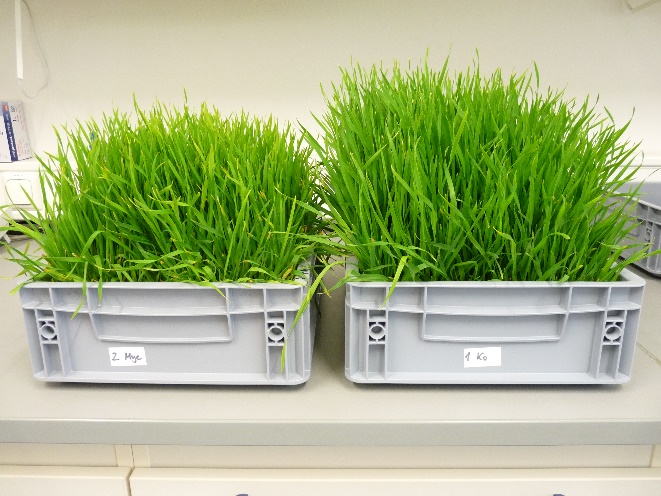

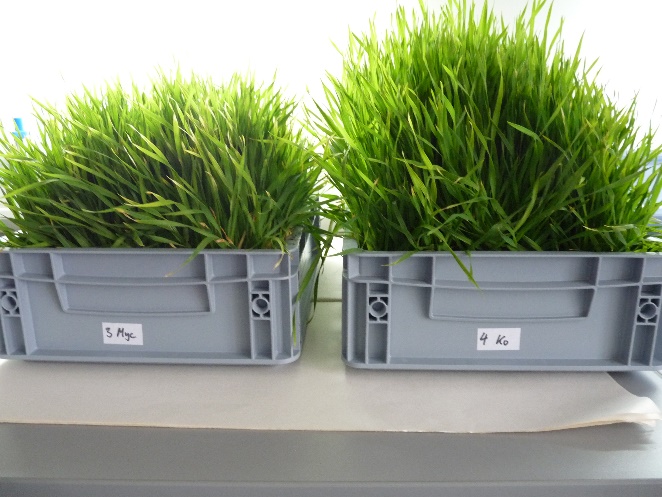


**Fig. S4.** Biomass development of winter wheat plants in a hydroponic system influenced by mycotoxins (left germination box) and the control (right germination box) after 8 days (left photo) and 14 days (right photo).

.


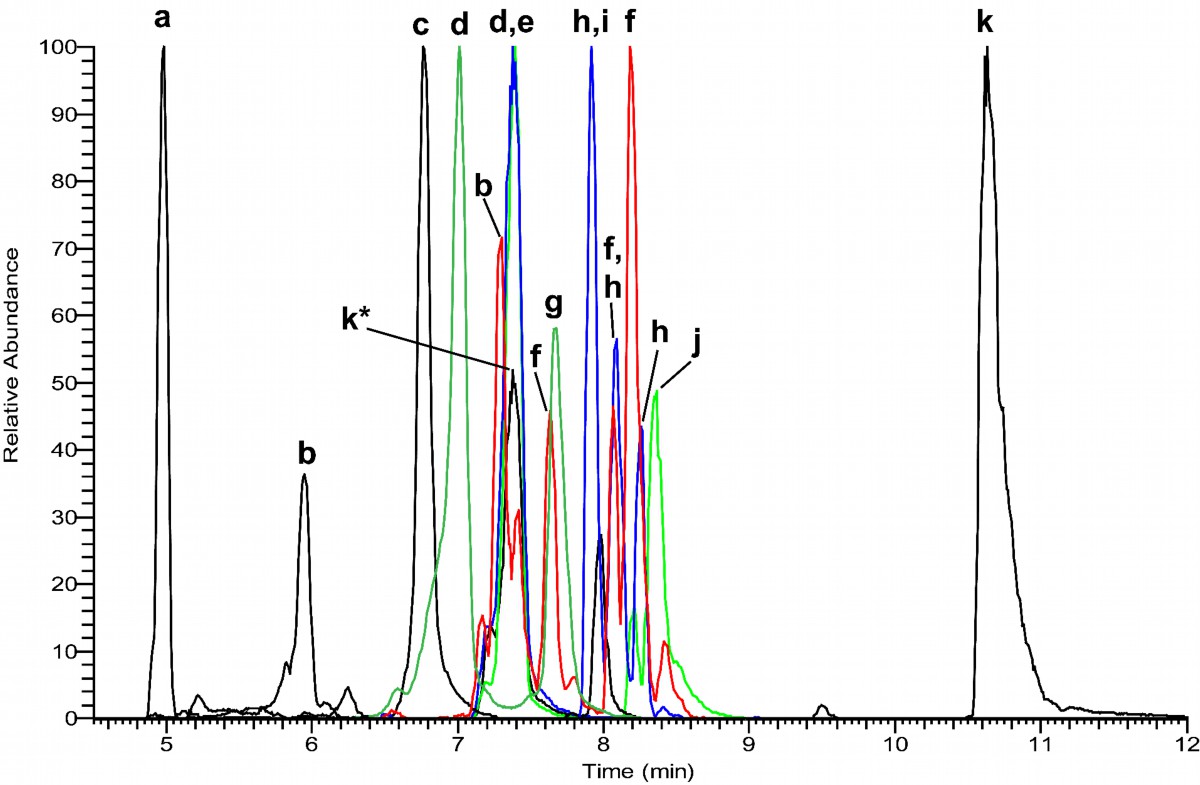


**Fig. S5.** Overlaid extracted ion chromatograms of alternariol (AOH) and its identified conjugates in root samples (abundances are normalized). a) AOH-3,9-disulfate, b) AOH-malonyldihexoside, c) AOH-3/9-sulfate-9/3-hexoside, d) AOH-acetylhexoside, e) AOH-6‘-malonyl-3-glucoside, f) AOH-dihexoside, g) AOH-3-sulfate, h) AOH-desoxyhexosylhexoside, i) AOH-pentosylhexoside, j) AOH-3-glucoside, k) AOH, k*) AOH in-source fragment of AOH-acetylhexoside and/or AOH-6‘-malonyl-3-glucoside.


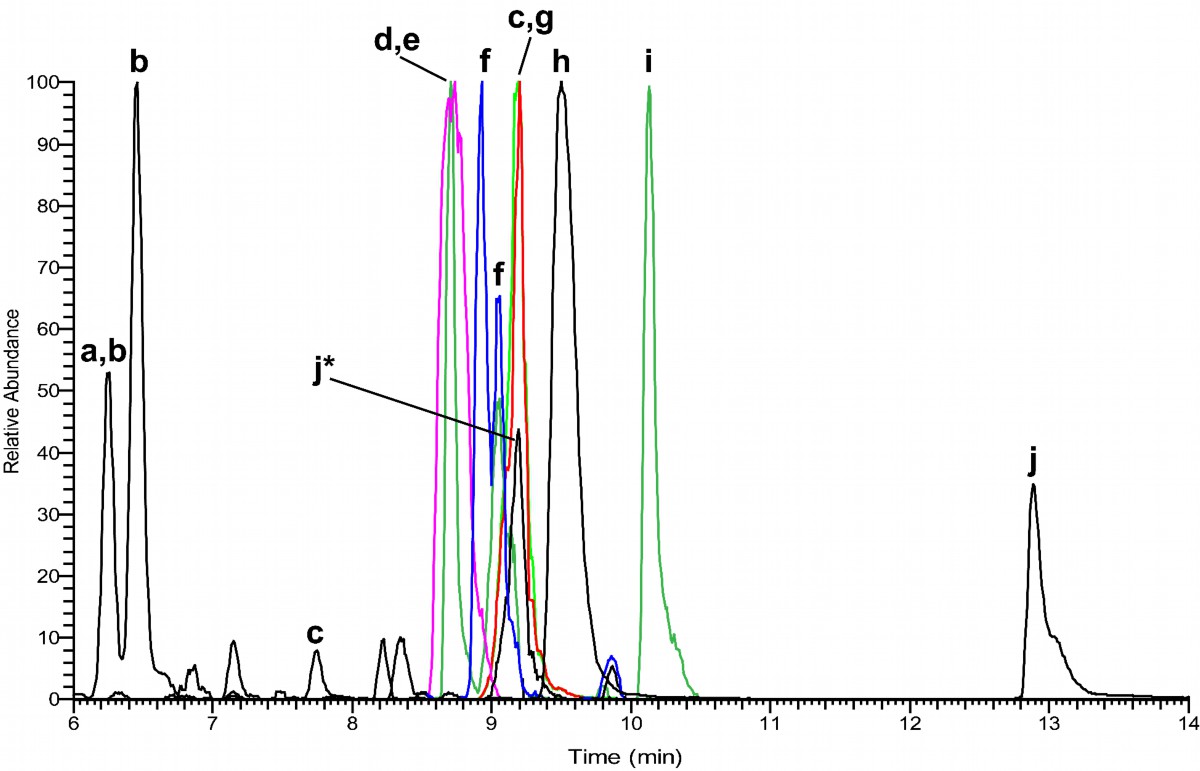


**Fig. S6.** Overlaid extracted ion chromatograms of alternariol-9-methylether (AME) and its identified conjugates in root samples (abundances are normalized). a) AME-trihexoside, b) AME-dihexoside, c) AME-acetylhexoside, d) AME-7-glucoside, e) AME-3/7-sulfate-7/3-glucoside, f) AME-malonyldihexoside, g) AME-6‘-malonyl-7-glucoside, h) AME-3-sulfate, i) AME-3-glucoside, j) AME, j*) AME in-source fragment of AME-acetylhexoside and/or AME-6‘-malonyl-7-glucoside.

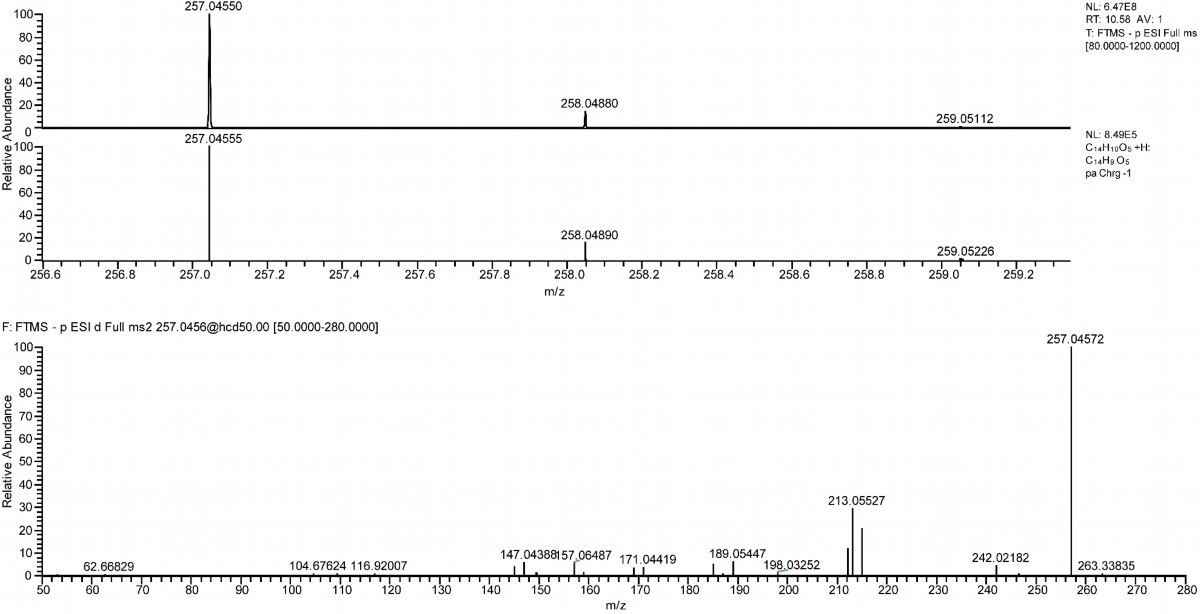


**a)**

**b)**

**Fig. S7.** HR-MS identification of alternariol (AOH, standard, peak #AOH-27). a) Comparison of the experimental (top) and theoretical (bottom) isotopic patterns. b) MS^2^ spectrum.

**a)**

**b)**


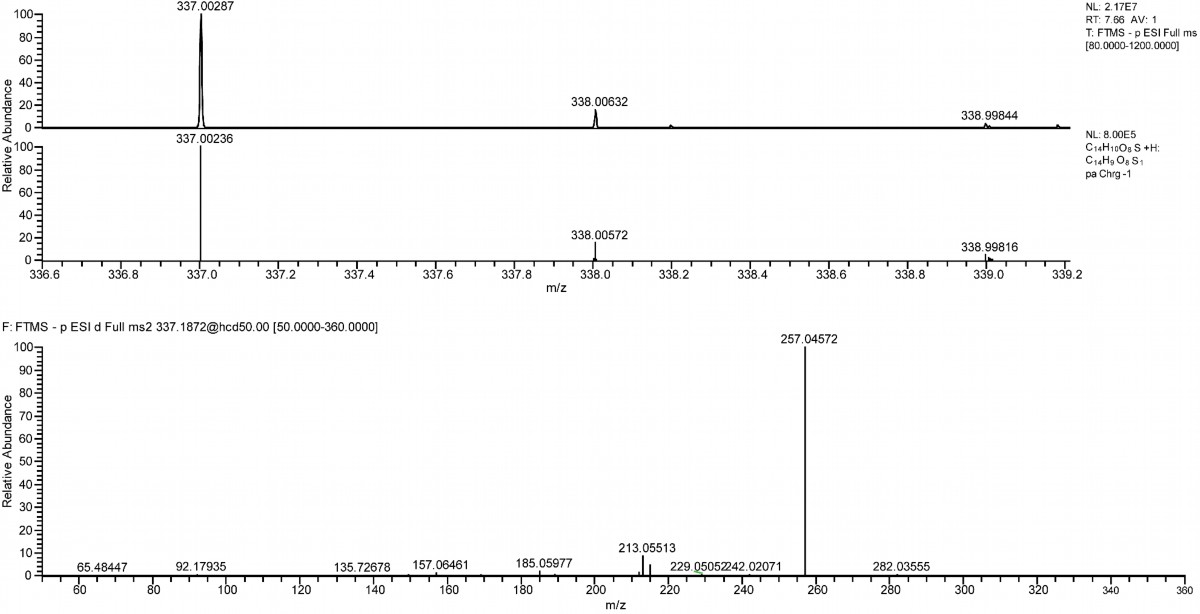


**Fig. S8.** HR-MS identification of AOH-3-sulfate (standard, peak #AOH-18). a) Comparison of the experimental (top) and theoretical (bottom) isotopic patterns. b) MS^2^ spectrum.

**a)**

**b)**


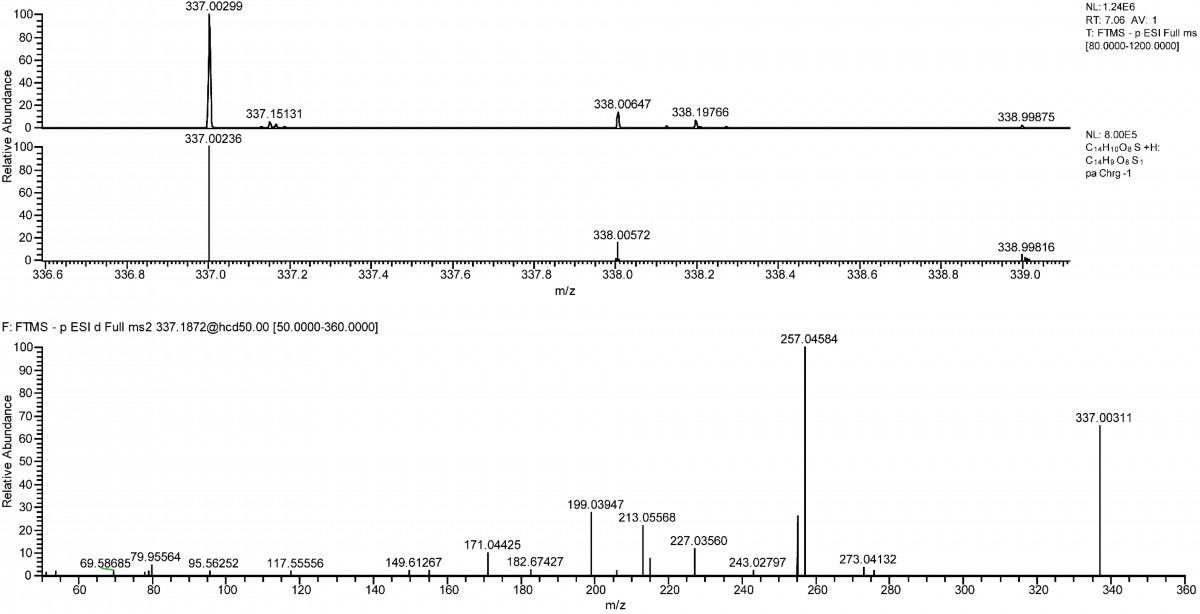


**Fig. S9.** HR-MS identification of AOH-sulfate (presumably AOH-9-sulfate, peak #AOH-11). a) Comparison of the experimental (top) and theoretical (bottom) isotopic patterns. b) MS^2^ spectrum. The structure is putative, actual positions may vary.

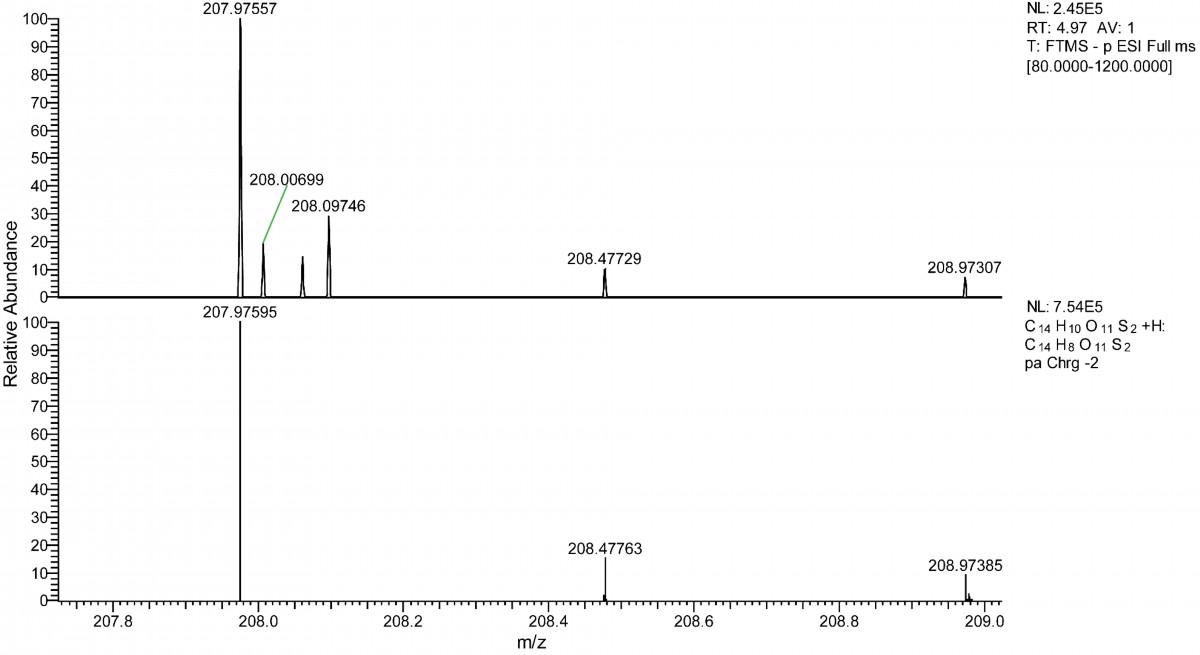


**Fig. S10.** HR-MS identification of AOH-disulfate (presumably AOH-3,9-disulfate, peak #AOH-1). Comparison of the experimental (top) and theoretical (bottom) isotopic patterns. The structure is putative, actual positions may vary.

**a)**

**b)**


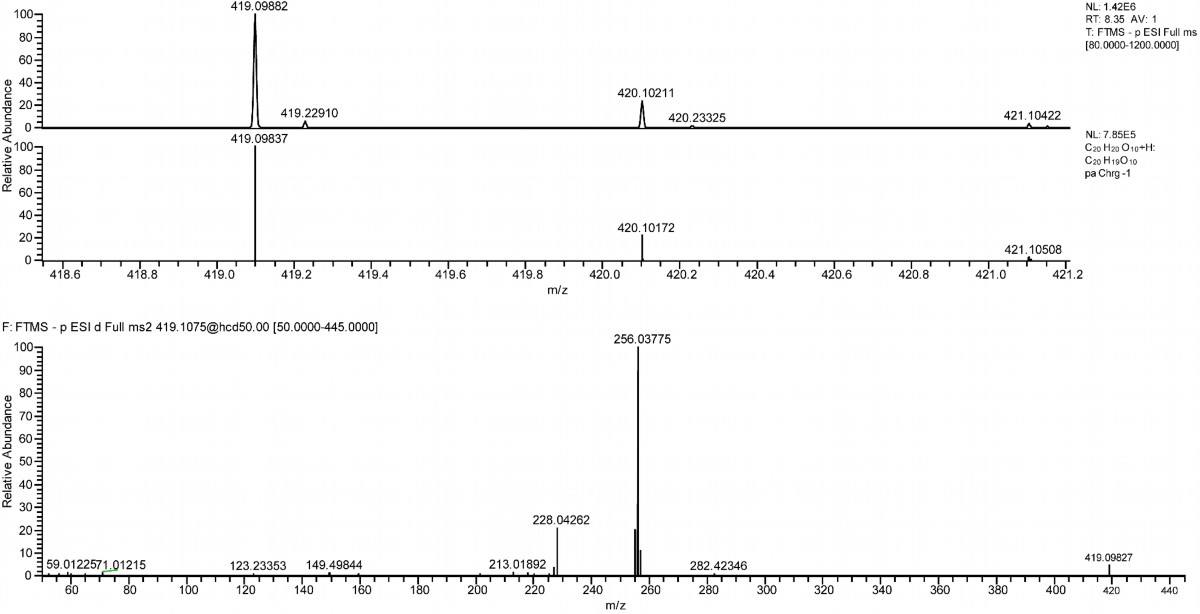


**Fig. S11.** HR-MS identification of AOH-3-glucoside (standard, peak #AOH-26). a) Comparison of the experimental (top) and theoretical (bottom) isotopic patterns. b) MS^2^ spectrum.

**a)**

**b)**


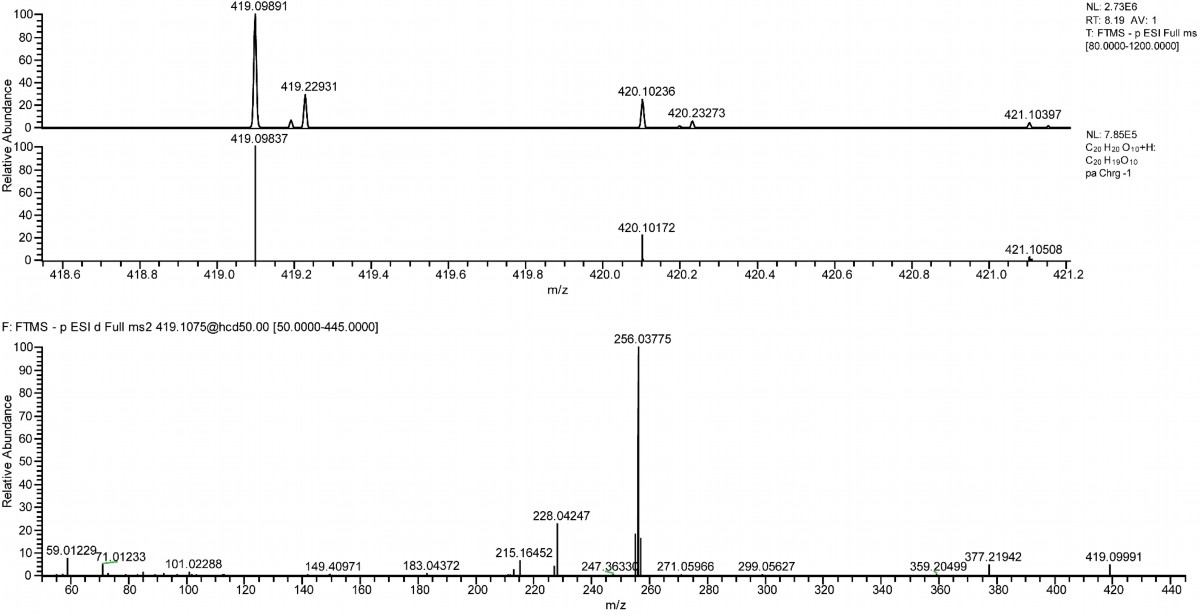


**Fig. S12.** HR-MS identification of AOH-9-glucoside (standard, peak #AOH-24). a) Comparison of the experimental (top) and theoretical (bottom) isotopic patterns. b) MS^2^ spectrum.

**a)**

**b)**


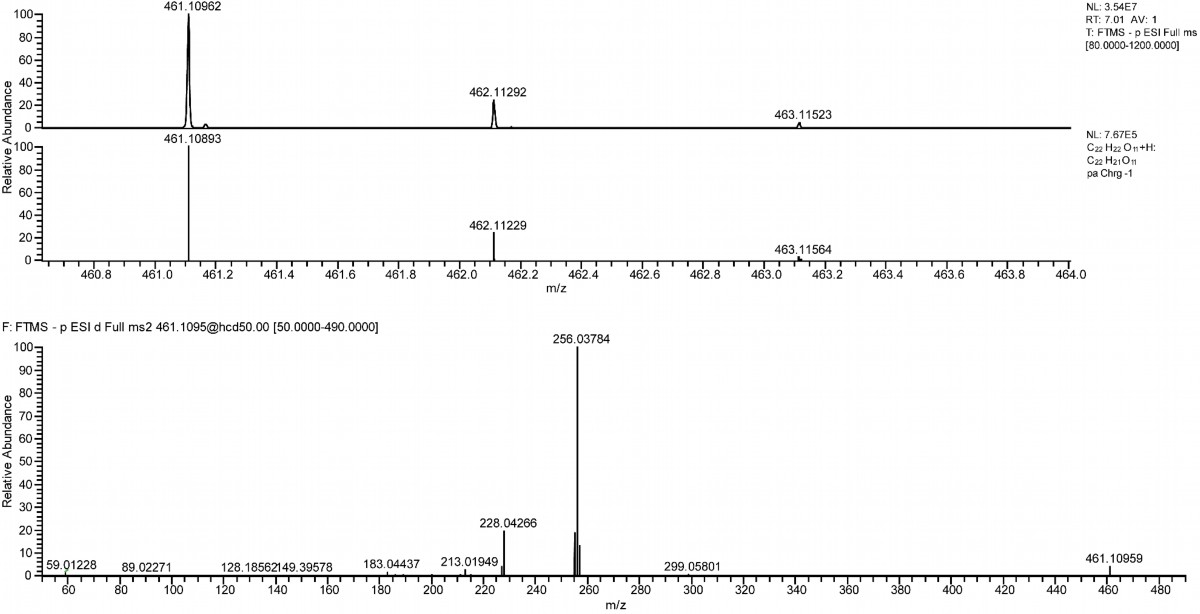


**Fig. S13.** HR-MS identification of AOH-acetylhexoside (presumably AOH-3-acetyl-9-hexoside, peak #AOH-10). a) Comparison of the experimental (top) and theoretical (bottom) isotopic patterns. b) MS^2^ spectrum. The structure is putative, actual positions may vary.

**a)**

**b)**


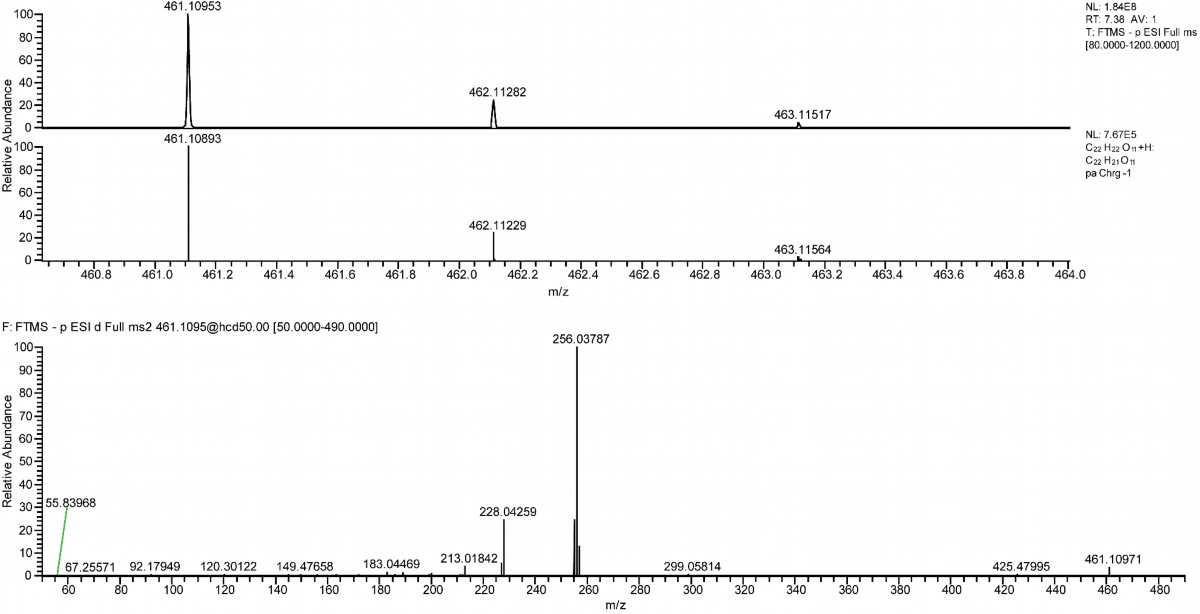


**Fig. S14.** HR-MS identification of AOH-acetylhexoside (presumably AOH-9-acetyl-3-hexoside, peak #AOH-13). a) Comparison of the experimental (top) and theoretical (bottom) isotopic patterns. b) MS^2^ spectrum. The structure is putative, actual positions may vary.

**a)**

**b)**


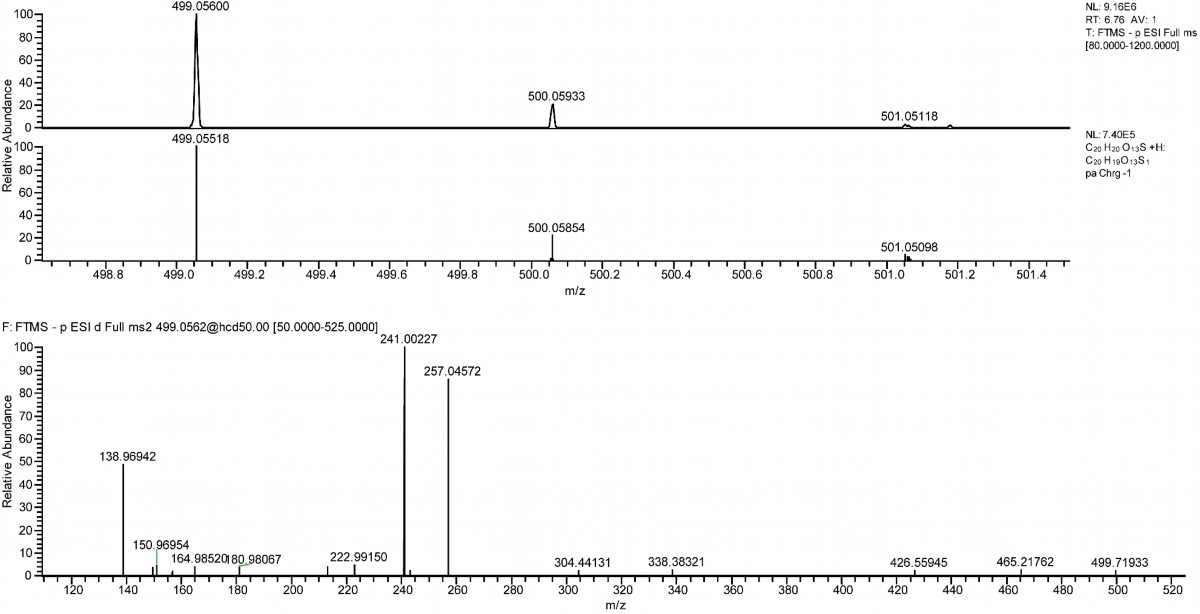


**Fig. S15.** HR-MS identification of AOH-sulfohexoside (presumably AOH-3-sulfate-9-hexoside, peak #AOH-8). a) Comparison of the experimental (top) and theoretical (bottom) isotopic patterns. b) MS^2^ spectrum. The structure is putative, actual positions may vary.

**a)**

**b)**


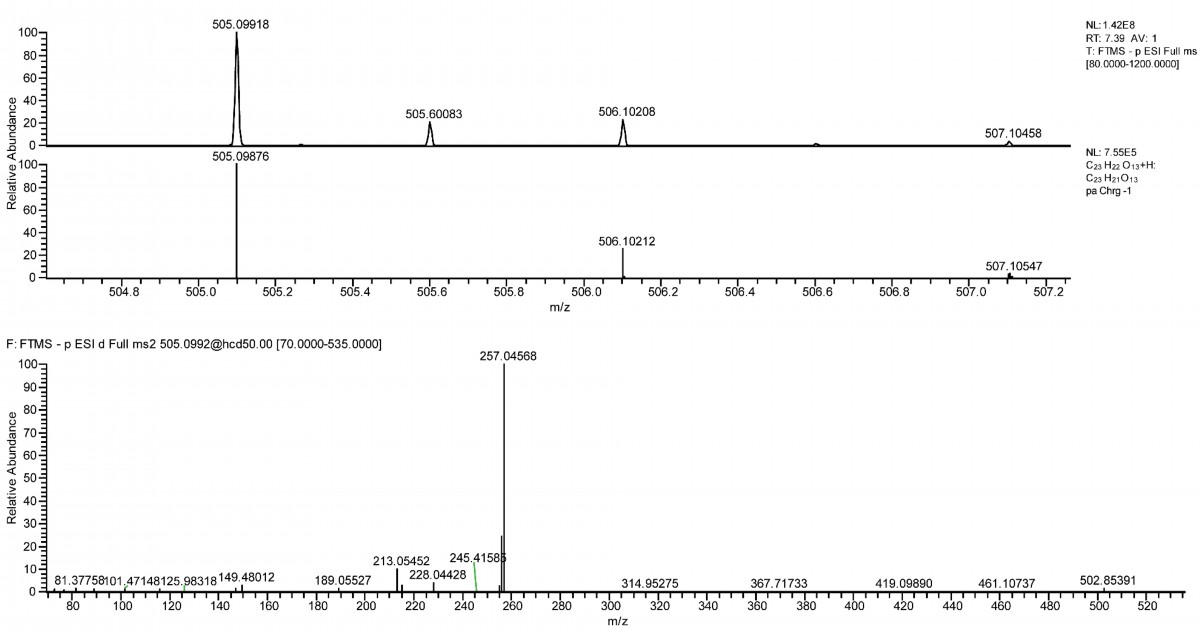


**Fig. S16.** HR-MS identification of AOH-6‘-malonyl-3-glucoside (standard, peak #AOH-14). a) Comparison of the experimental (top) and theoretical (bottom) isotopic patterns. b) MS^2^ spectrum.

**a)**

**b)**


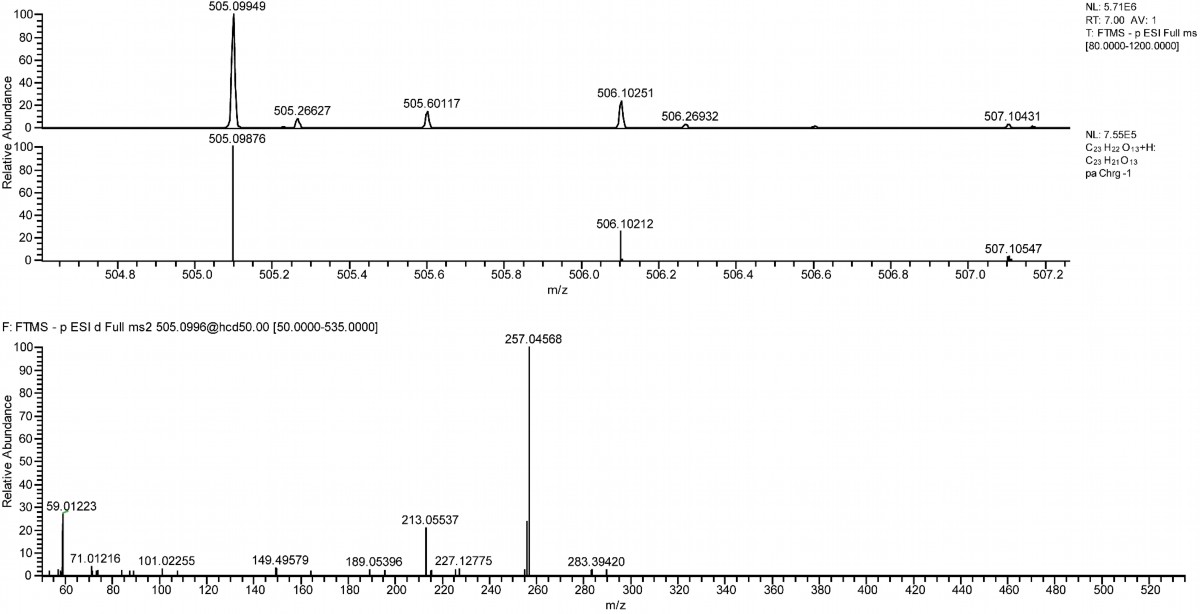


**Fig. S17.** HR-MS identification of AOH-6‘-Malonyl-9-glucoside (standard, peak #AOH-9). a) Comparison of the experimental (top) and theoretical (bottom) isotopic patterns. b) MS^2^ spectrum.

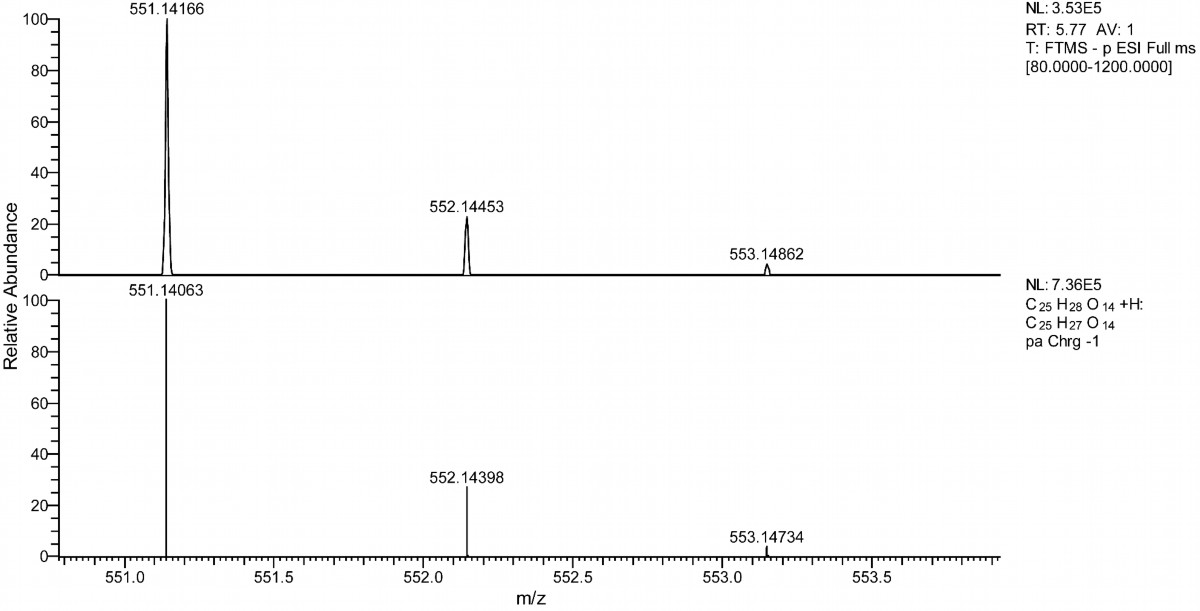


**Fig. S18.** HR-MS identification of AOH-pentosylhexoside (presumably AOH-3-pentosyl-9-hexoside, peak #AOH-3). Comparison of the experimental (top) and theoretical (bottom) isotopic patterns. The structure is putative, actual positions may vary.

**a)**

**b)**


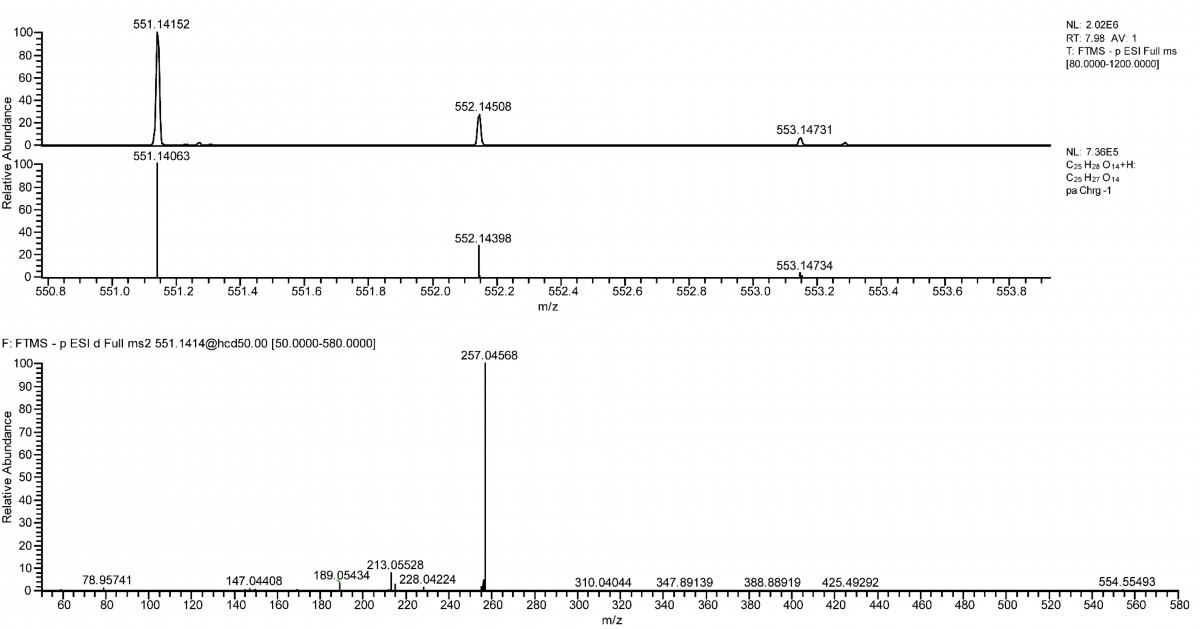


**Fig. S19.** HR-MS identification of AOH-pentosylhexoside (presumably AOH-3-pentosylhexoside, peak #AOH-20). a) Comparison of the experimental (top) and theoretical (bottom) isotopic patterns. b) MS^2^ spectrum. The structure is putative, actual positions may vary.

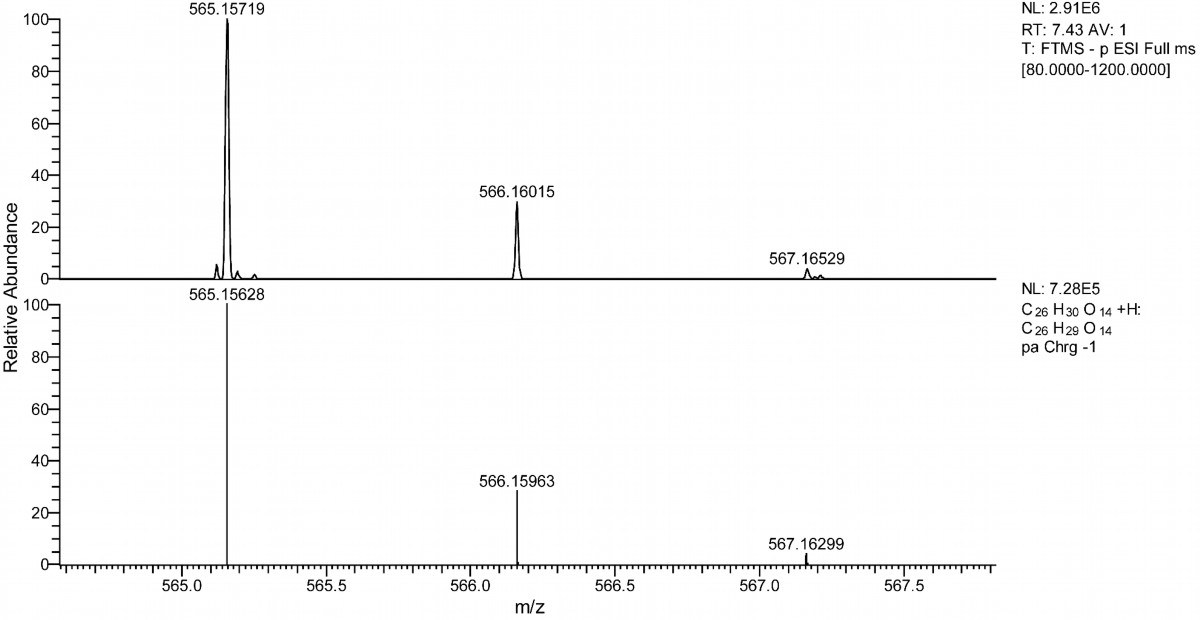


**Fig. S20.** HR-MS identification of AOH-desoxyhexosylhexoside (presumably AOH-3-desoxyhexosyl-9-hexoside, peak #AOH-15). Comparison of the experimental (top) and theoretical (bottom) isotopic patterns. The structure is putative, actual positions may vary.

**a)**

**b)**


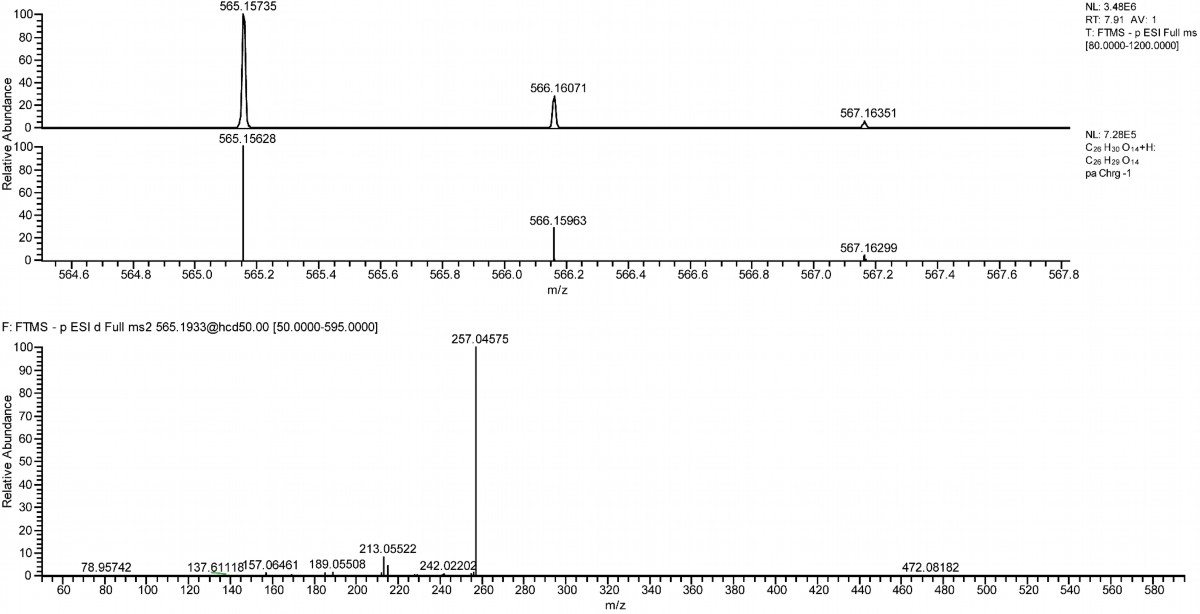


**Fig. S21.** HR-MS identification of AOH-desoxyhexosylhexoside (presumably AOH-9-desoxyhexosyl-3-hexoside, peak #AOH-19). a) Comparison of the experimental (top) and theoretical (bottom) isotopic patterns. b) MS^2^ spectrum. The structure is putative, actual positions may vary.

**a)**

**b)**

**Fig. S22.** HR-MS identification of AOH-desoxyhexosylhexoside (presumably AOH-9-desoxyhexosylhexoside, peak #AOH-22). a) Comparison of the experimental (top) and theoretical (bottom) isotopic patterns. b) MS^2^ spectrum. The structure is putative, actual positions may vary.


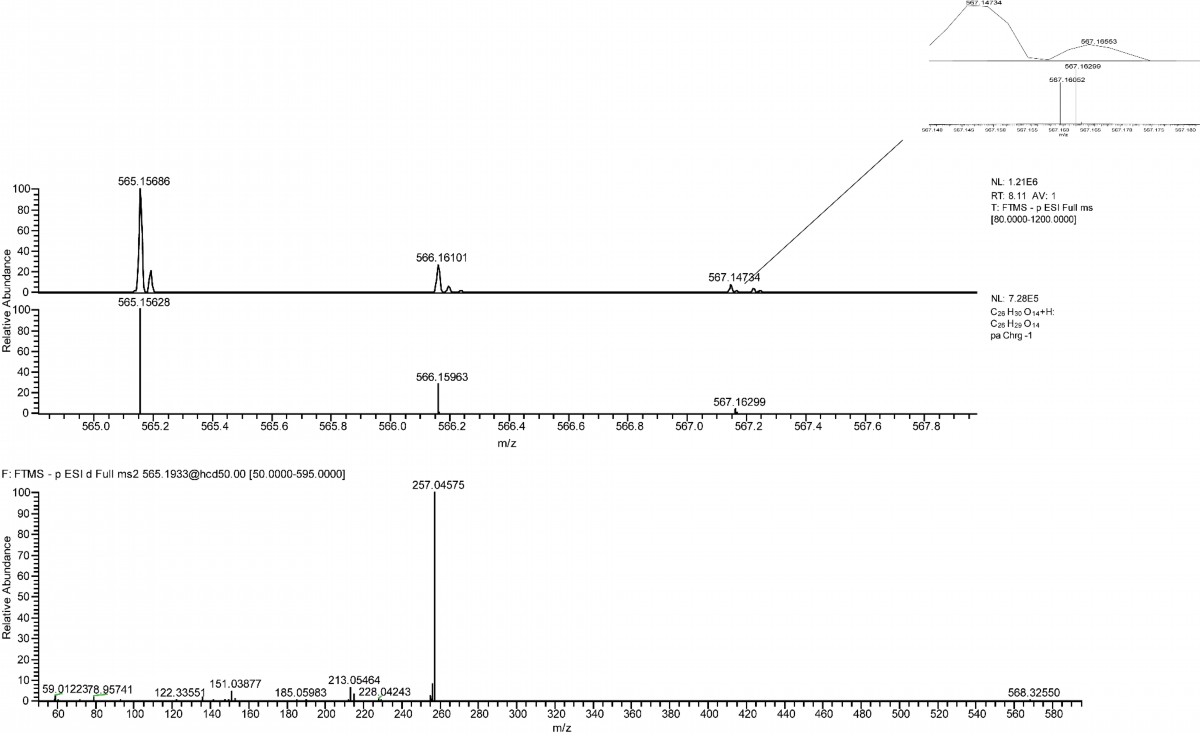

**a)**

**b)**


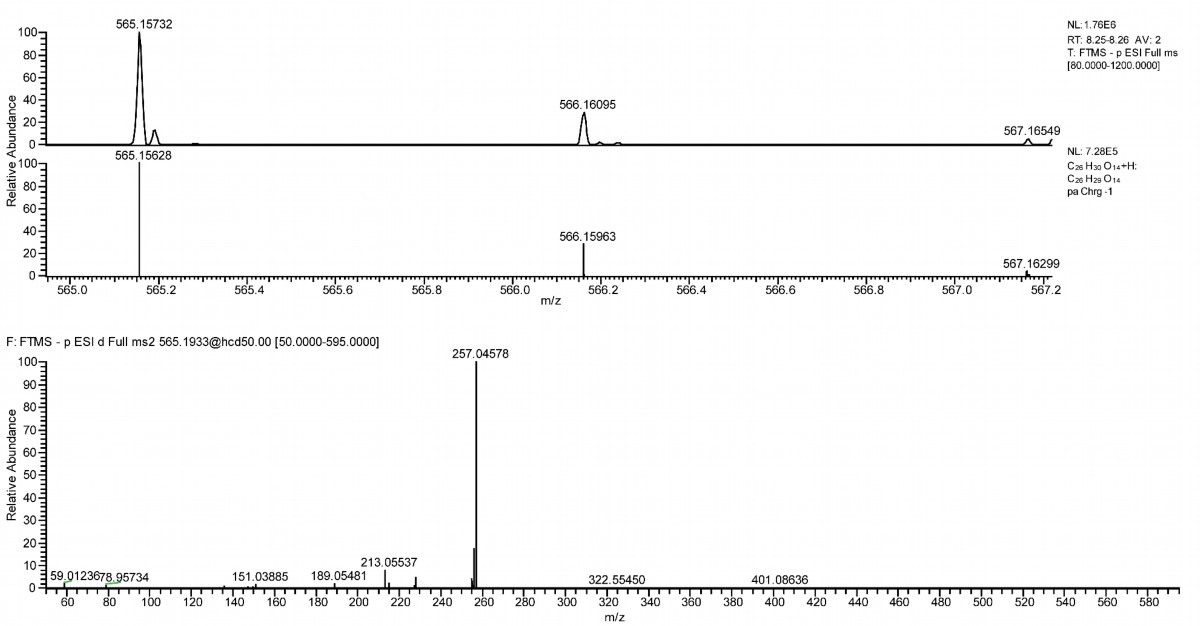


**Fig. S23.** HR-MS identification of AOH-desoxyhexosylhexoside (presumably AOH-3-desoxyhexosylhexoside, peak #AOH-25). a) Comparison of the experimental (top) and theoretical (bottom) isotopic patterns. b) MS^2^ spectrum. The structure is putative, actual positions may vary.

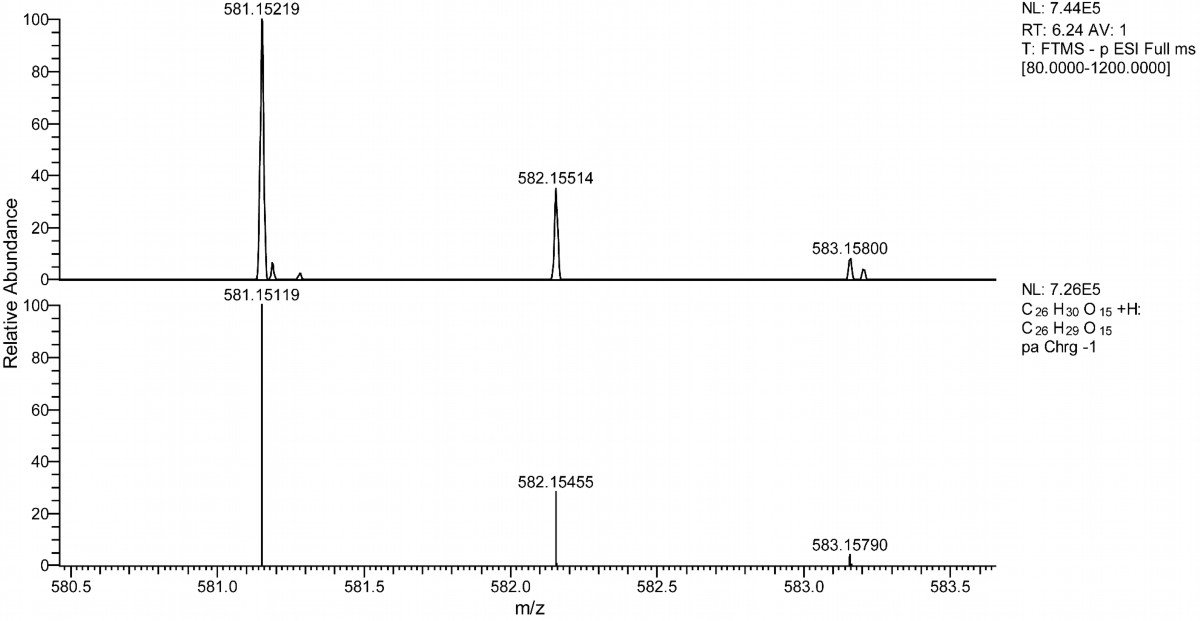


**Fig. S24.** HR-MS identification of AOH-dihexoside (presumably AOH-3,9-dihexoside, peak #AOH-6). Comparison of the experimental (top) and theoretical (bottom) isotopic patterns. The structure is putative, actual positions may vary.

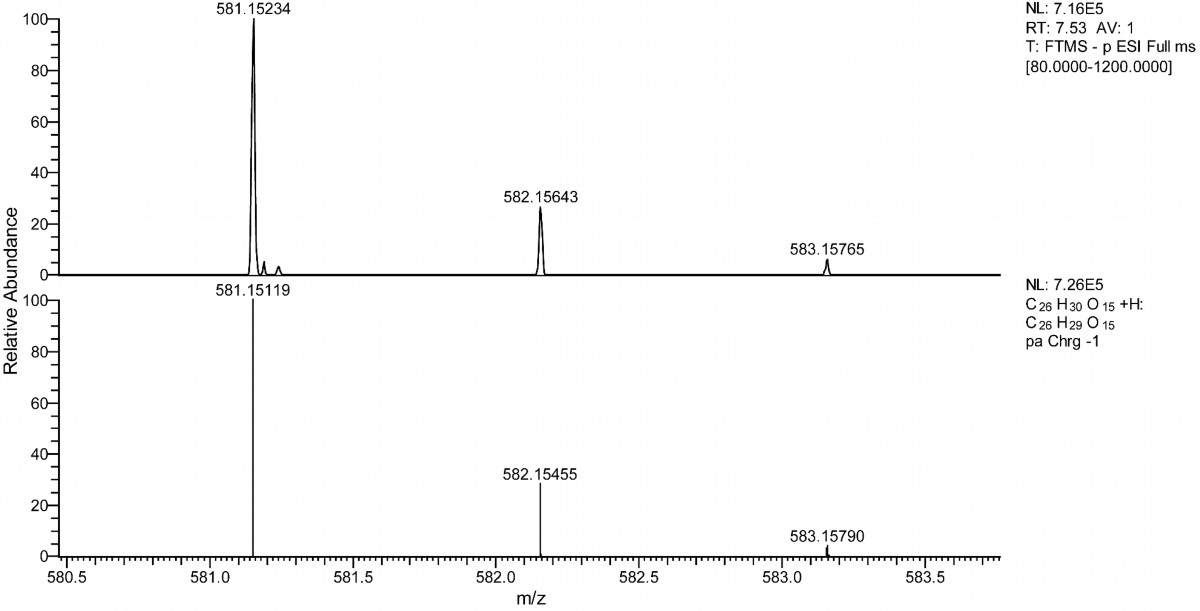


**Fig. S25.** HR-MS identification of AOH-9-diglucoside (standard, peak #AOH-16). Comparison of the experimental (top) and theoretical (bottom) isotopic patterns.

**a)**

**b)**


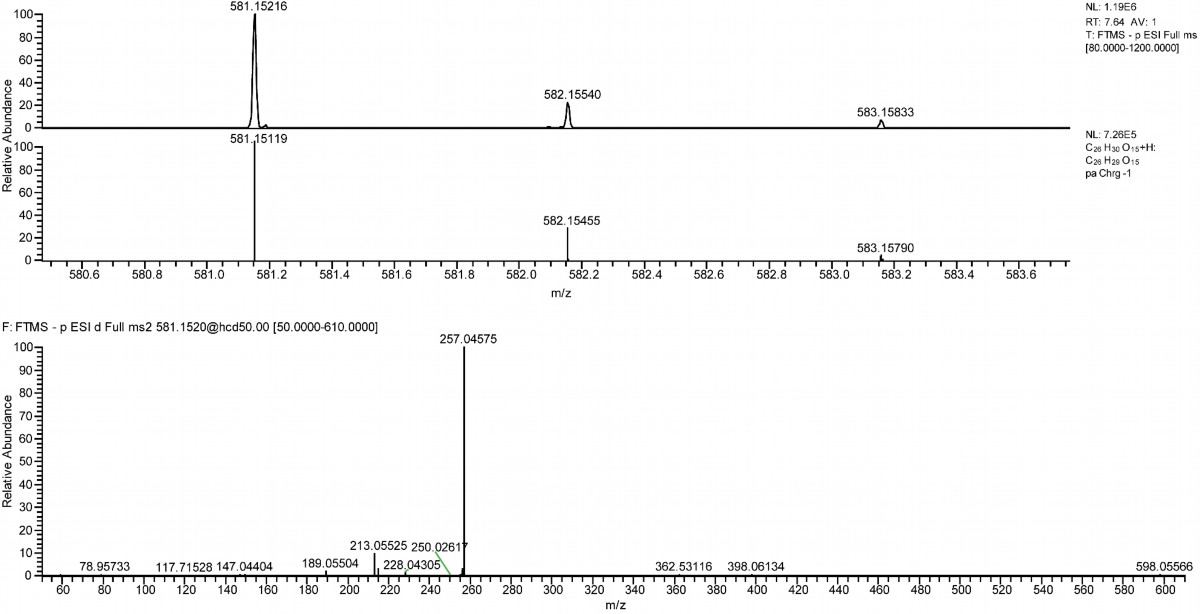


**Fig. S26.** HR-MS identification of AOH-dihexoside (presumably AOH-9-dihexoside, peak #AOH-17). a) Comparison of the experimental (top) and theoretical (bottom) isotopic patterns. b) MS^2^ spectrum. The structure is putative, actual positions may vary.

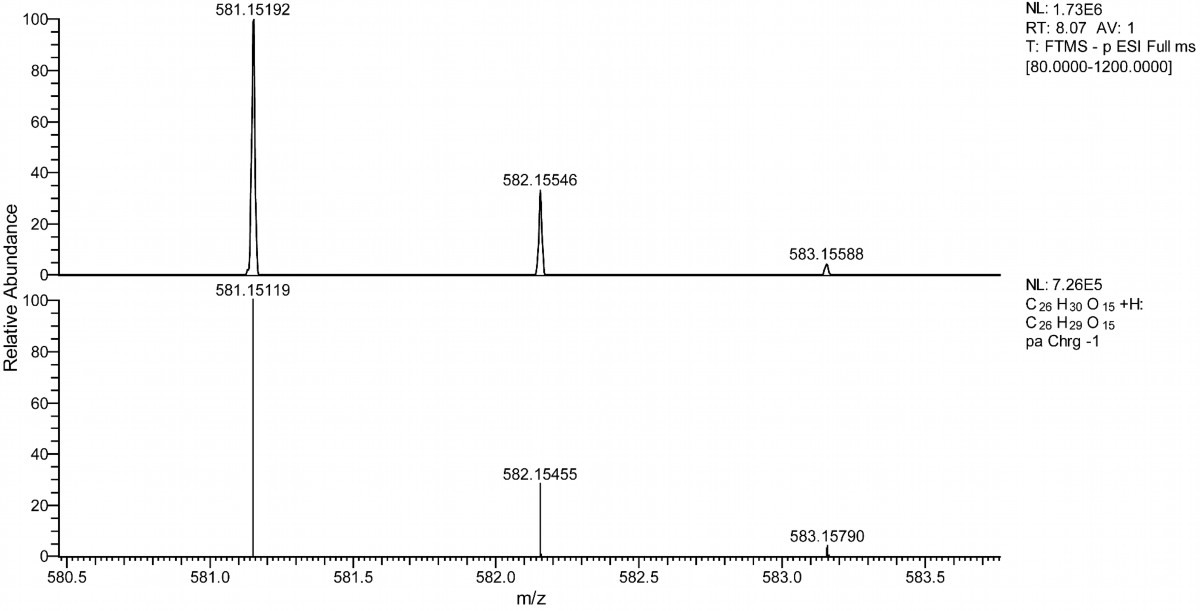


**Fig. S27.** HR-MS identification of AOH-dihexoside (presumably AOH-3-dihexoside, peak #AOH-21). Comparison of the experimental (top) and theoretical (bottom) isotopic patterns. The structure is putative, actual positions may vary.

**a)**

**b)**


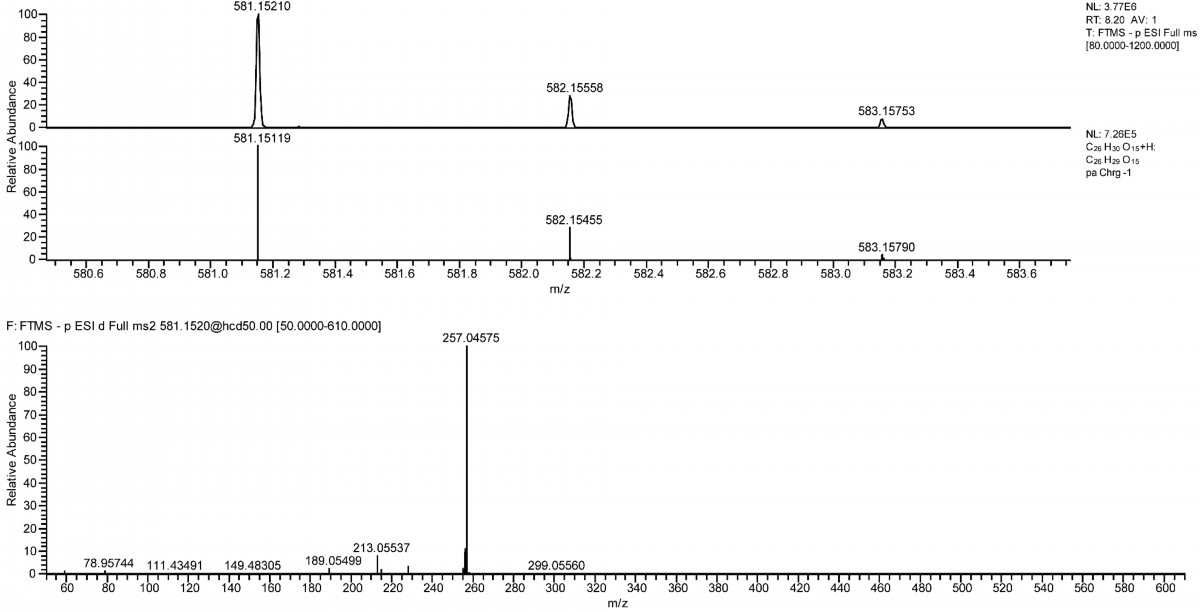


**Fig. S28.** HR-MS identification of AOH-dihexoside (presumably AOH-3-dihexoside, peak #AOH-23). a) Comparison of the experimental (top) and theoretical (bottom) isotopic patterns. b) MS^2^ spectrum. The structure is putative, actual positions may vary.

**Fig. S29.** HR-MS identification of AOH-malonyldihexoside (presumably AOH-3-hexosyl-9-malonylhexoside, peak #AOH-2). Comparison of the experimental (top) and theoretical (bottom) isotopic patterns. The structure is putative, actual positions may vary.


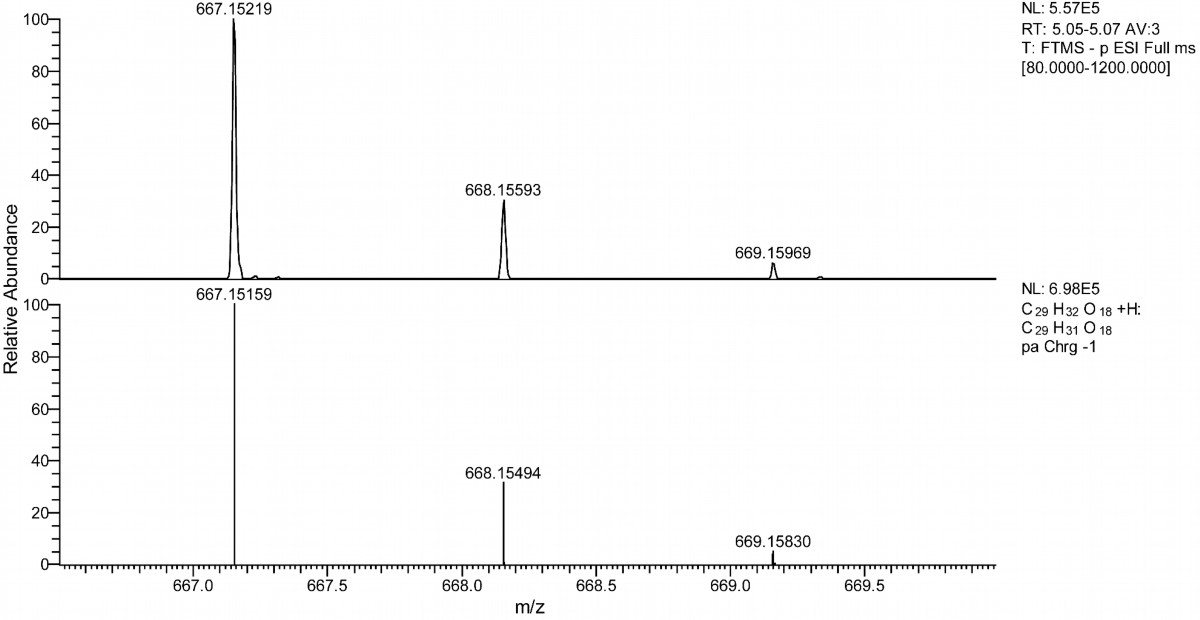

**a)**

**b)**


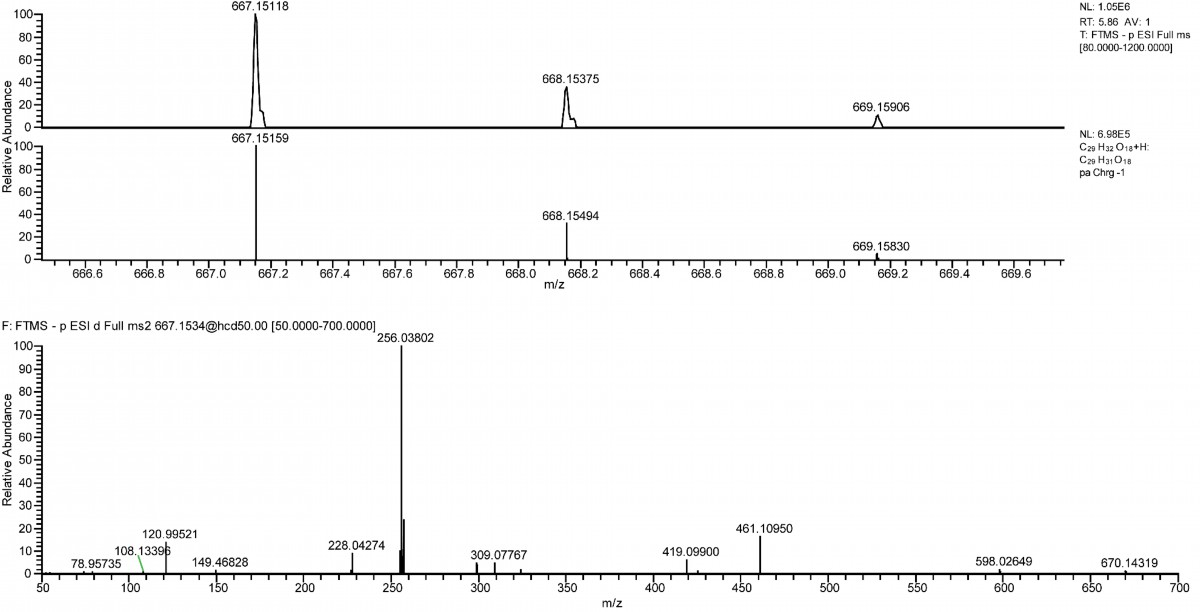


**Fig. S30.** HR-MS identification of AOH-malonyldihexoside (presumably AOH-9-hexosyl-3-malonylhexoside, peak #AOH-5). a) Comparison of the experimental (top) and theoretical (bottom) isotopic patterns. b) MS^2^ spectrum. The structure is putative, actual positions may vary.

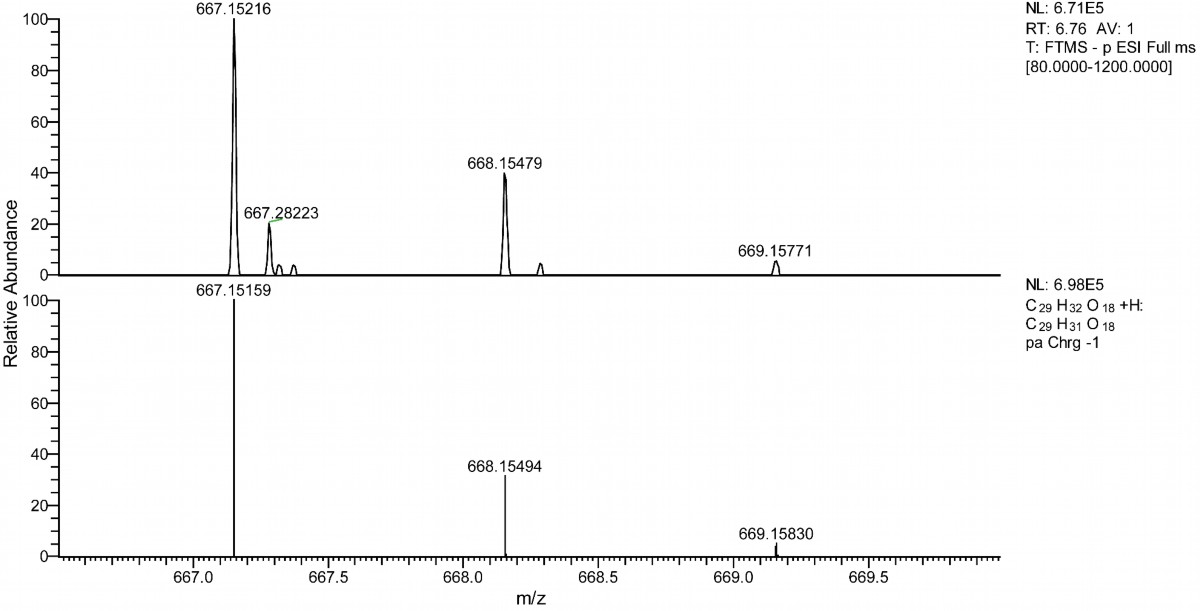


**Fig. S31.** HR-MS identification of AOH-malonyldihexoside (presumably AOH-9-malonyldihexoside, peak #AOH-7). Comparison of the experimental (top) and theoretical (bottom) isotopic patterns. The structure is putative, actual positions may vary.

**a)**

**b)**


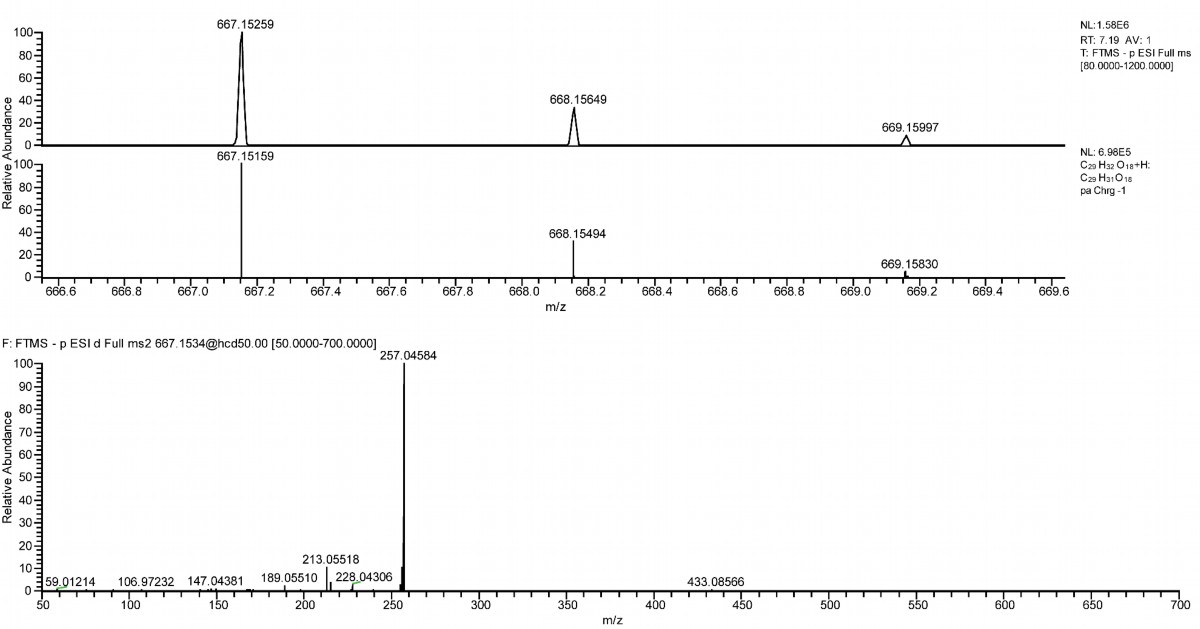


**Fig. S32.** HR-MS identification of AOH-malonyldihexoside (presumably AOH-3-malonyldihexoside, peak #AOH-12). a) Comparison of the experimental (top) and theoretical (bottom) isotopic patterns. b) MS^2^ spectrum. The structure is putative, actual positions may vary.

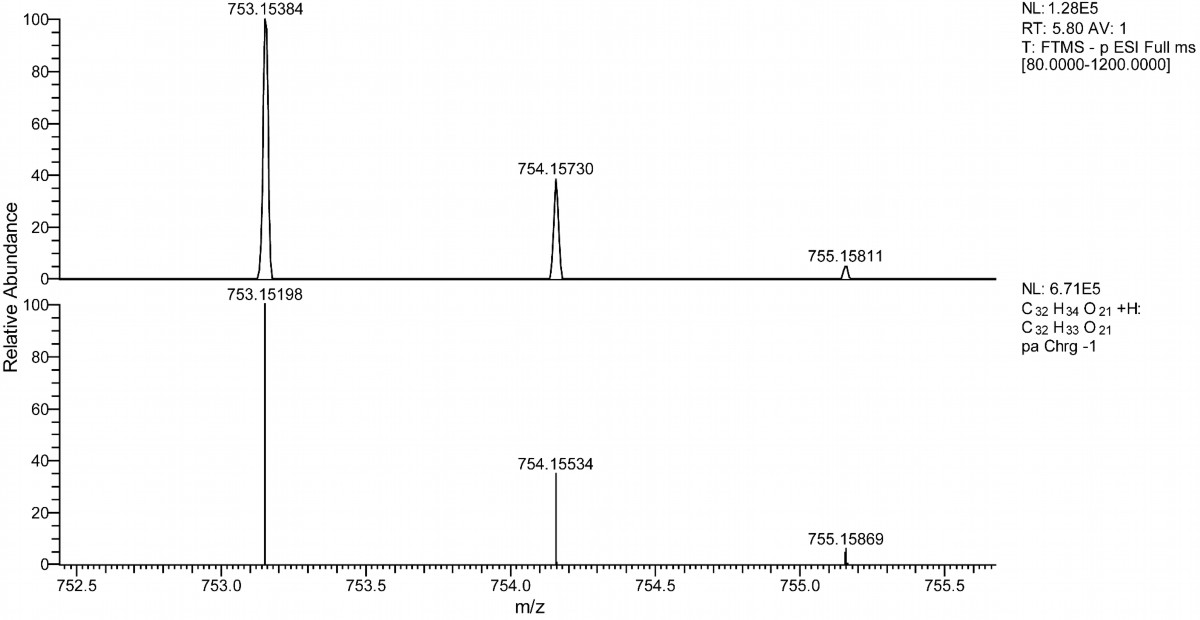


**Fig. S33.** HR-MS identification of AOH-dimalonyldihexoside (presumably AOH-3,9-dimalonyldihexoside, peak #AOH-4). Comparison of the experimental (top) and theoretical (bottom) isotopic patterns. The structure is putative, actual positions may vary.

**a)**

**b)**


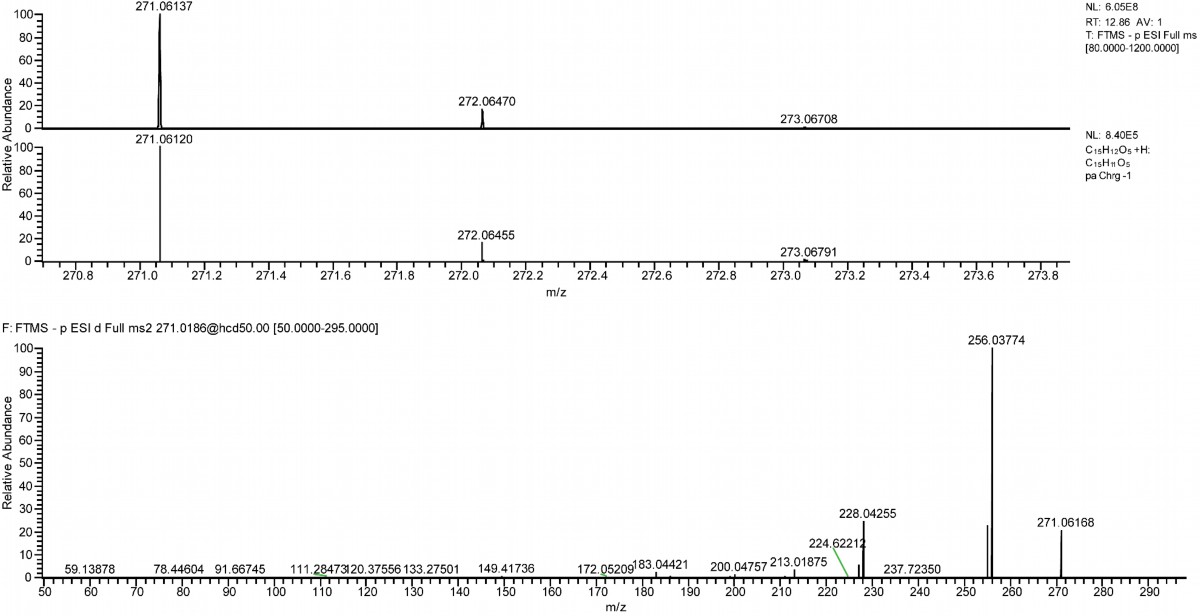


**Fig. S34.** HR-MS identification of AOH-9-O-monomethyl ether (AME, standard, peak #AME-19). a) Comparison of the experimental (top) and theoretical (bottom) isotopic patterns. b) MS^2^ spectrum.

**a)**

**b)**


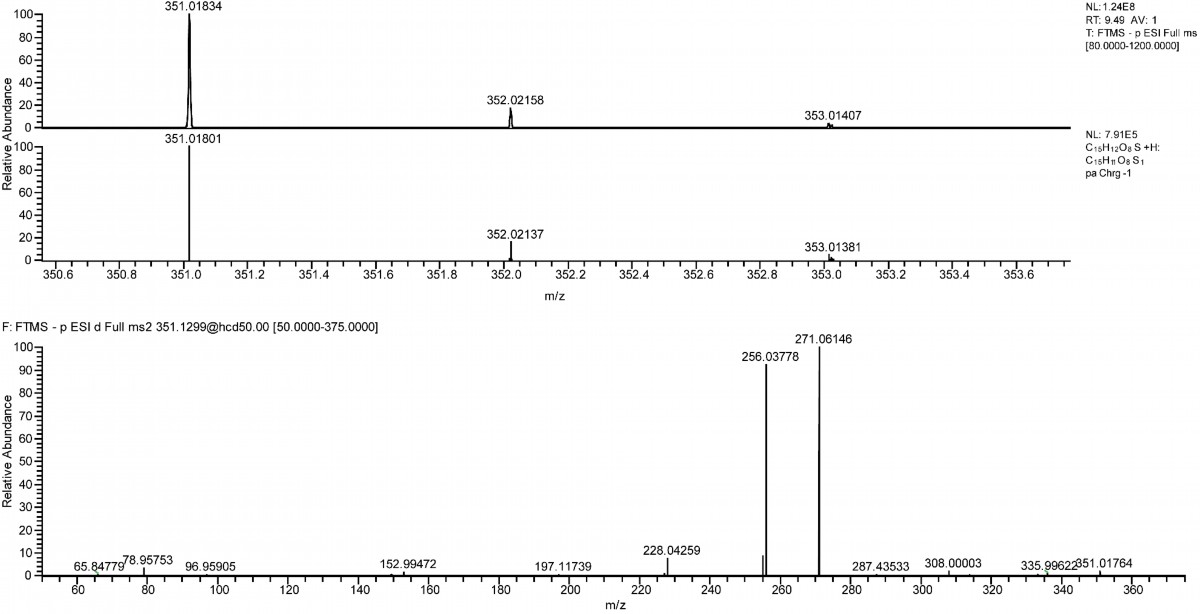


**Fig. S35.** HR-MS identification of AME-3-sulfate (standard, peak #AME-16). a) Comparison of the experimental (top) and theoretical (bottom) isotopic patterns. b) MS^2^ spectrum.

**a)**

**b)**


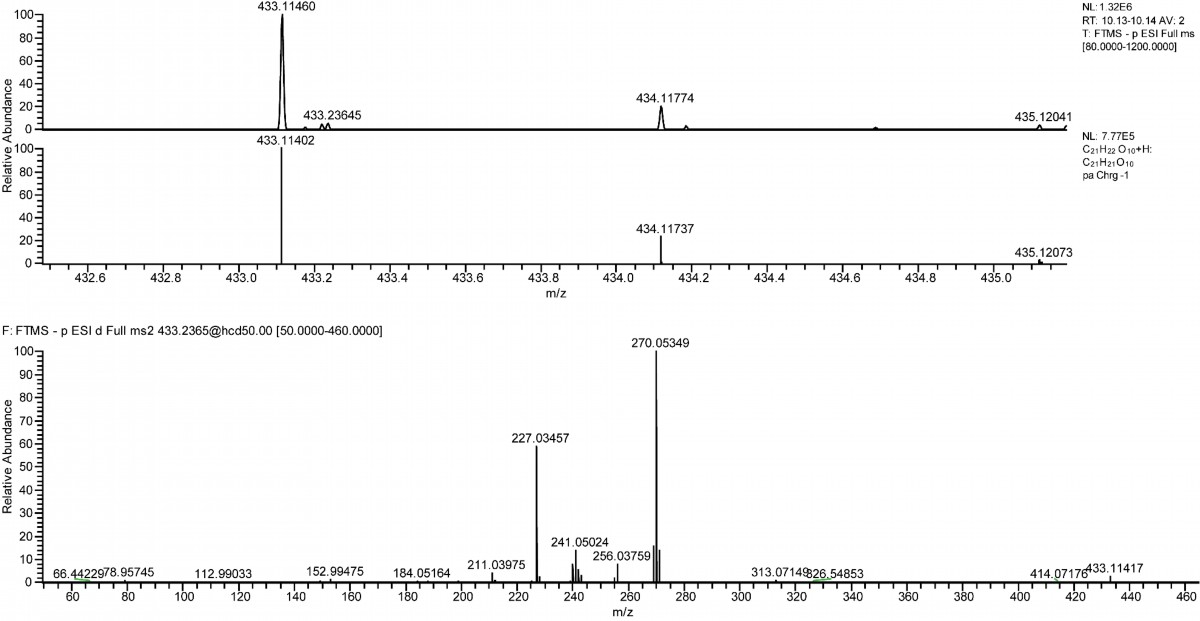


**Fig. S36.** HR-MS identification of AME-3-glucoside (standard, peak #AME-17). a) Comparison of the experimental (top) and theoretical (bottom) isotopic patterns. b) MS^2^ spectrum.

**a)**

**b)**

**Fig. S37.** HR-MS identification of AME-7-glucoside (standard, peak #AME-8). a) Comparison of the experimental (top) and theoretical (bottom) isotopic patterns. b) MS^2^ spectrum.


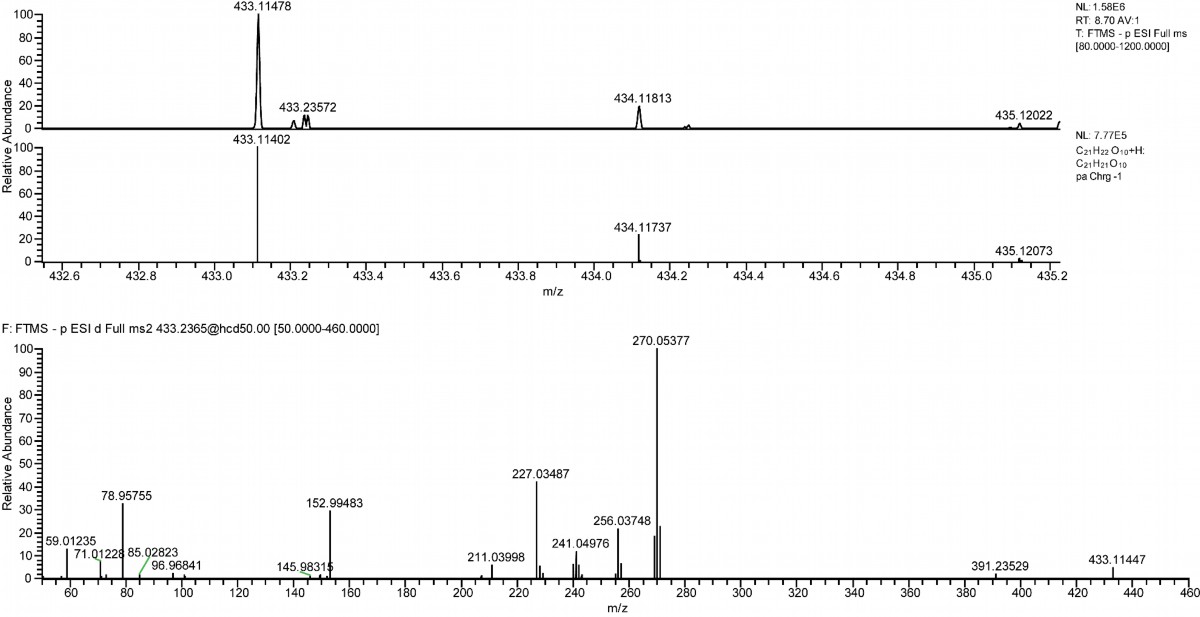

**a)**

**b)**


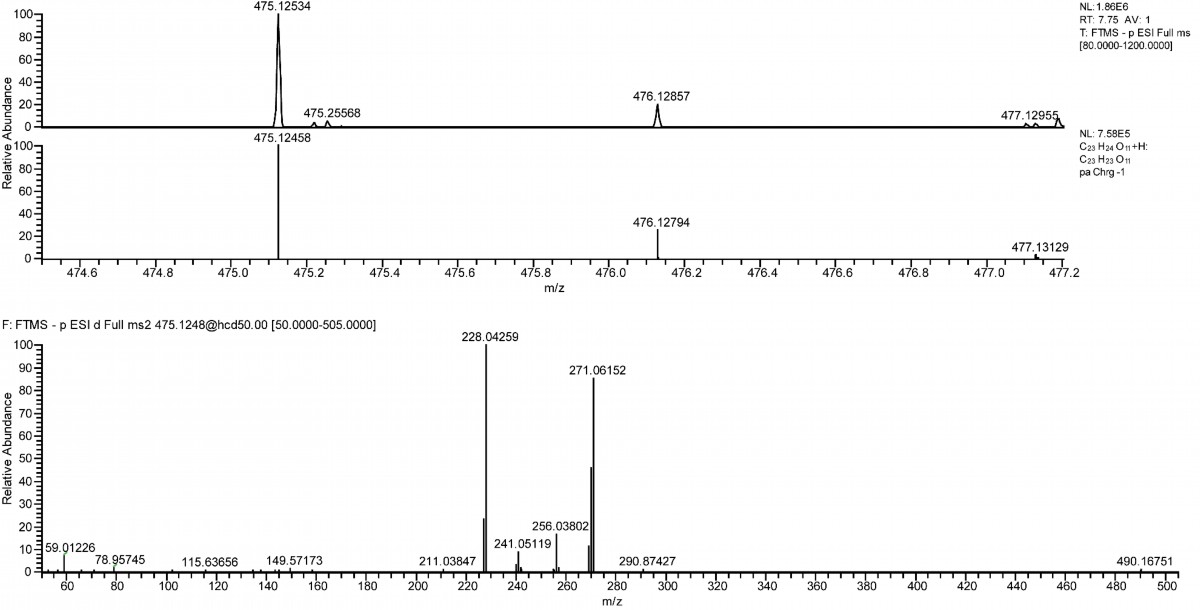


**Fig. S38.** HR-MS identification of AME-acetylhexoside (presumably AME-7-acetylhexoside, peak #AME-7). a) Comparison of the experimental (top) and theoretical (bottom) isotopic patterns. b) MS^2^ spectrum. The structure is putative, actual positions may vary.

**a)**

**b)**


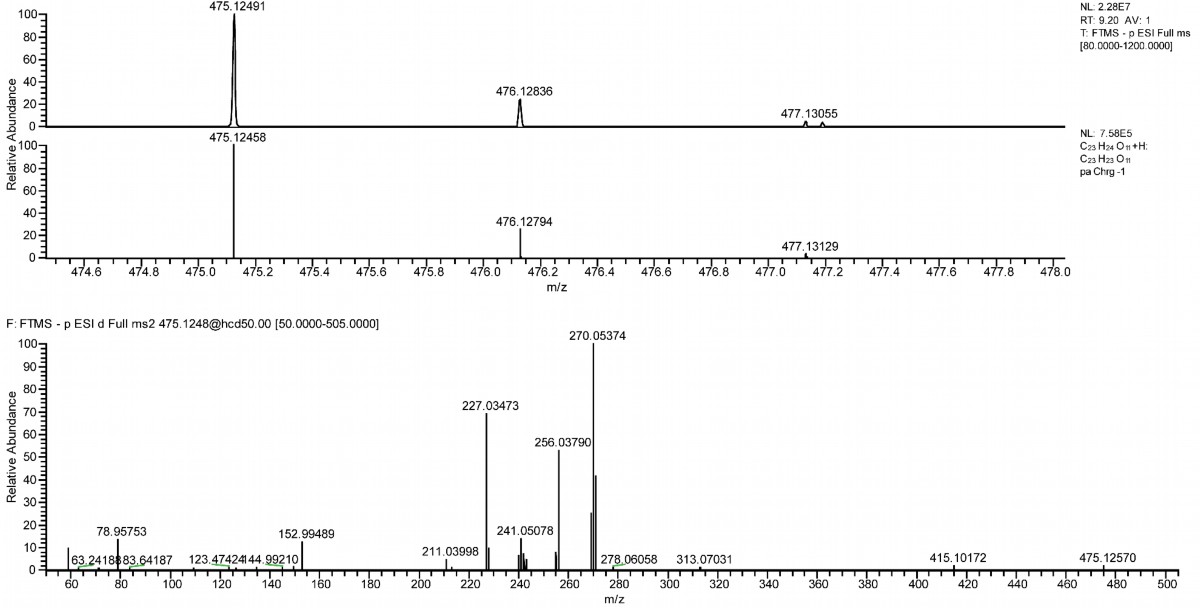


**Fig. S39.** HR-MS identification of AME-acetylhexoside (presumably AME-7-acetyl-3-hexoside, peak #AME-13). a) Comparison of the experimental (top) and theoretical (bottom) isotopic patterns. b) MS^2^ spectrum. The structure is putative, actual positions may vary.

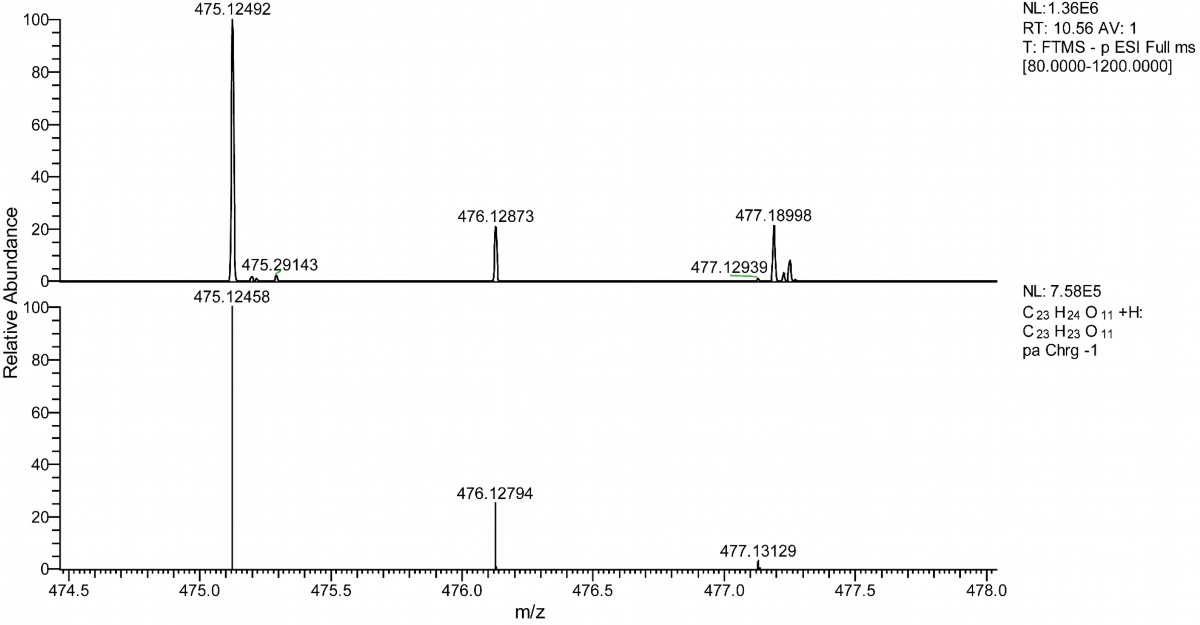


**Fig. S40.** HR-MS identification of AME-acetylhexoside (presumably AME-3-acetylhexoside, peak #AME-18). Comparison of the experimental (top) and theoretical (bottom) isotopic patterns. The structure is putative, actual positions may vary.

**a)**

**b)**


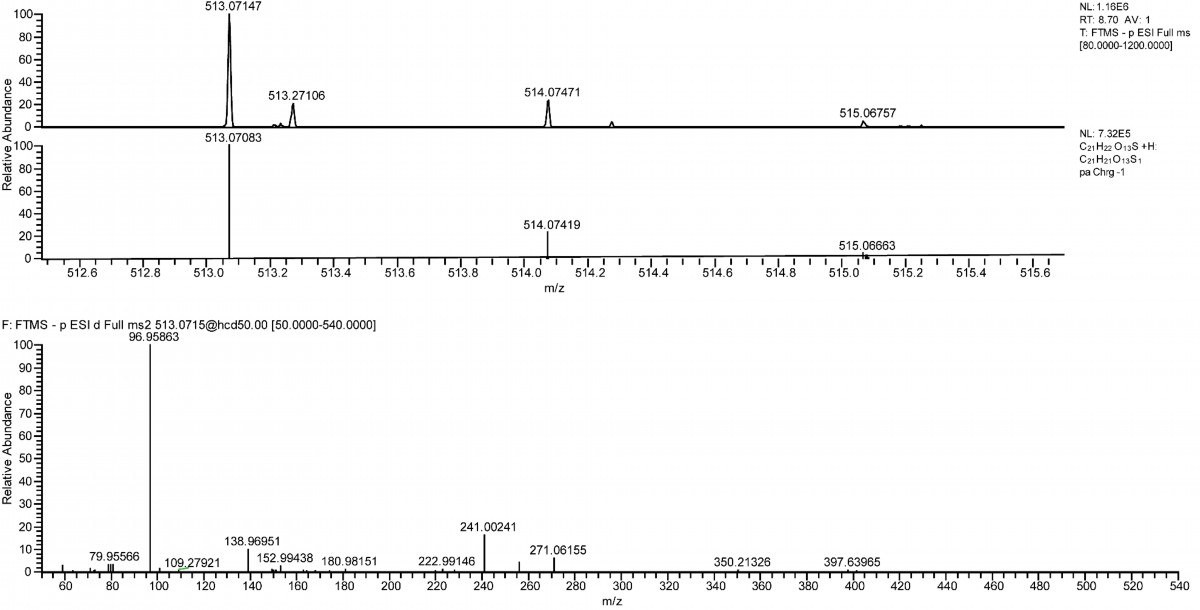


**Fig. S41.** HR-MS identification of AME-sulfohexoside (presumably AME-3-sulfate-7-hexoside, peak #AME-8). a) Comparison of the experimental (top) and theoretical (bottom) isotopic patterns. b) MS^2^ spectrum. The structure is putative, actual positions may vary.

**a)**

**b)**


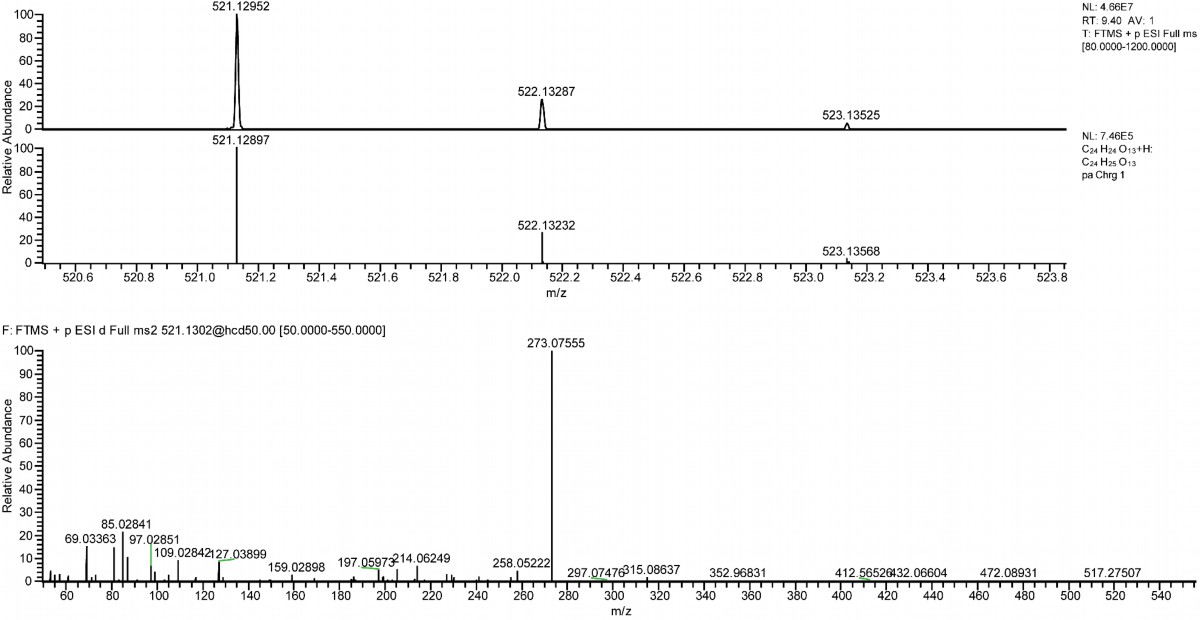


**Fig. S42.** HR-MS identification of AME-6‘-malonyl-3-glucoside (standard, peak #AME-15, positive mode). a) Comparison of the experimental (top) and theoretical (bottom) isotopic patterns. b) MS^2^ spectrum.

**a)**

**b)**


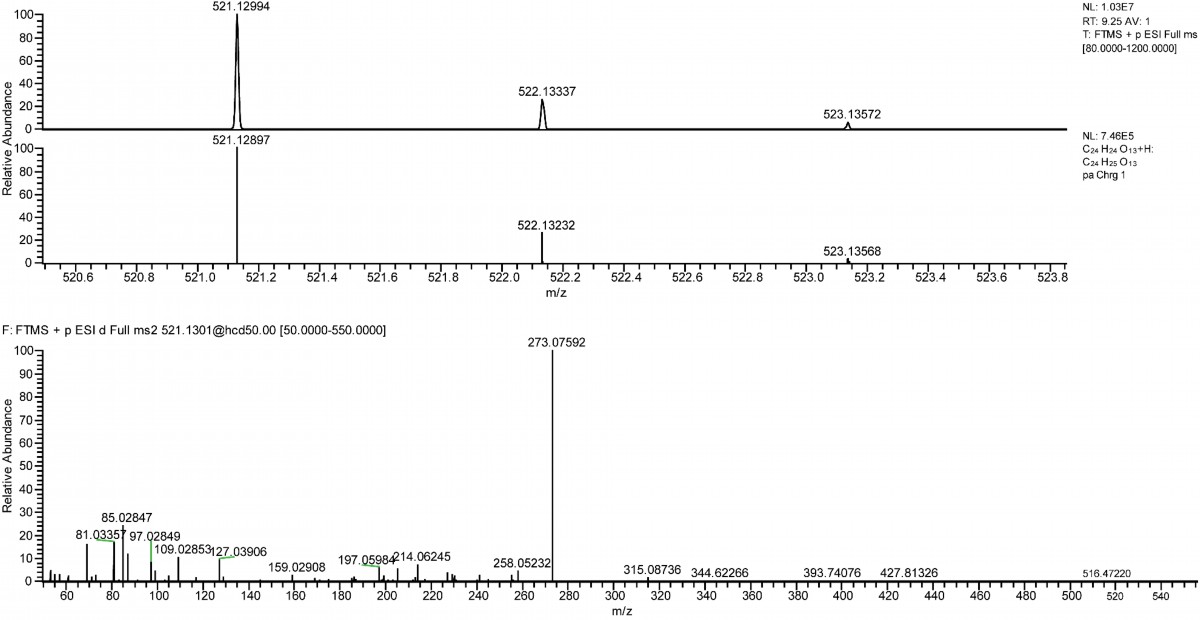


**Fig. S43.** HR-MS identification of AME-malonylhexoside (presumably AME-6‘-malonyl-7-hexoside, peak #AME-14, positive mode). a) Comparison of the experimental (top) and theoretical (bottom) isotopic patterns. b) MS^2^ spectrum. The structure is putative, actual positions may vary.

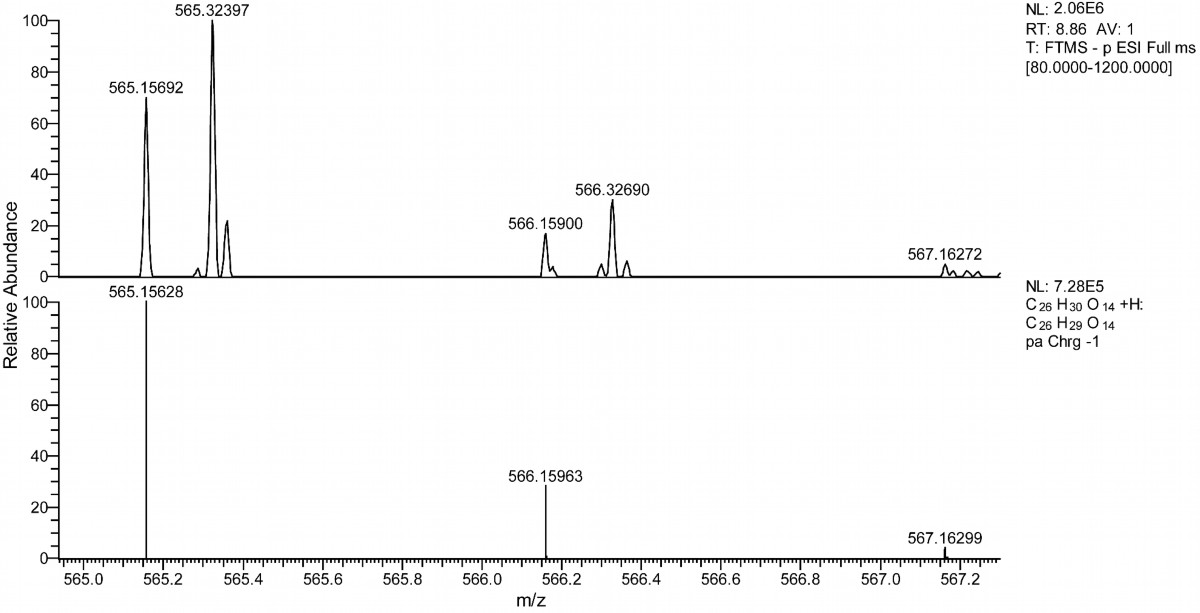


**Fig. S44.** HR-MS identification of AME-pentosylhexoside (presumably AME-3-pentosylhexoside, peak #AME-10). Comparison of the experimental (top) and theoretical (bottom) isotopic patterns. The structure is putative, actual positions may vary.

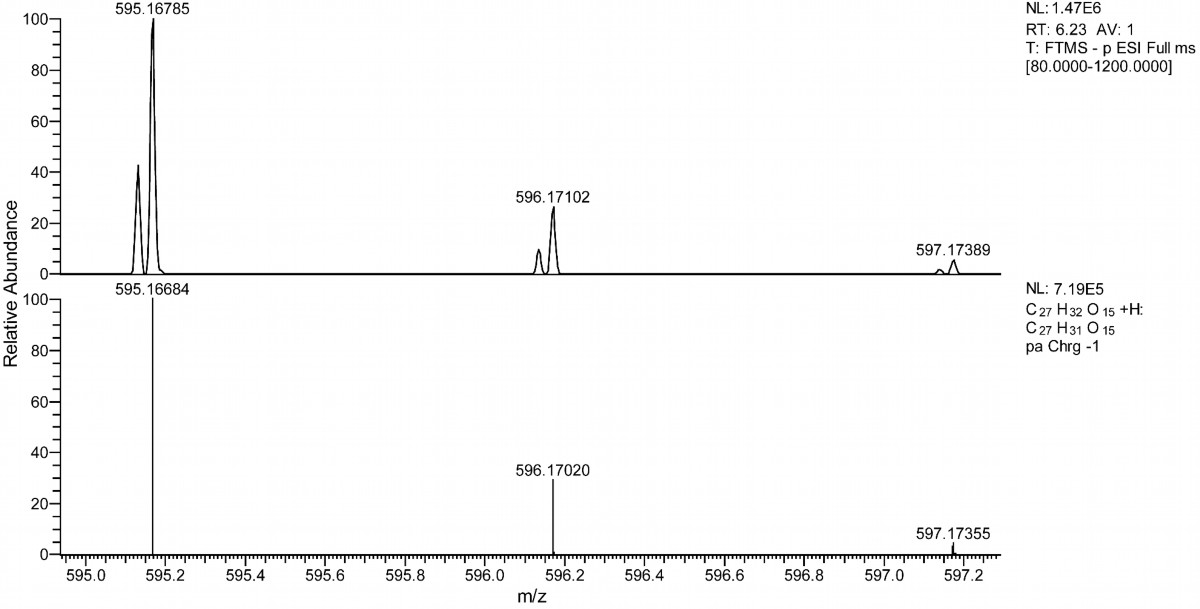


**Fig. S45.** HR-MS identification of AME-dihexoside (presumably AME-7-dihexoside, peak #AME-2). Comparison of the experimental (top) and theoretical (bottom) isotopic patterns. The structure is putative, actual positions may vary.

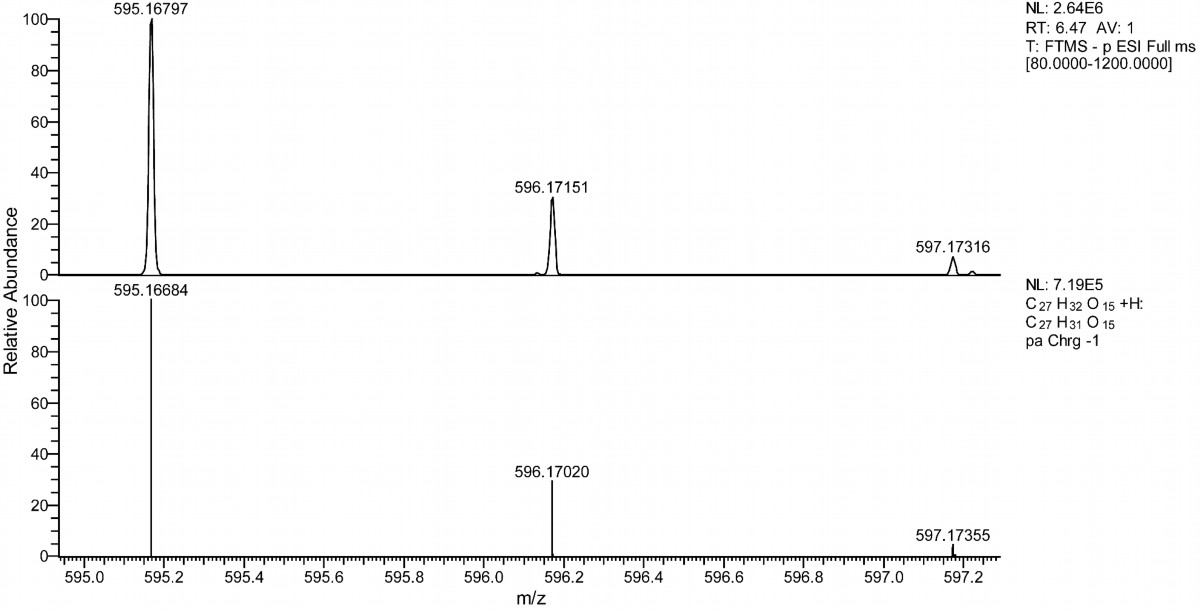


**Fig. S46.** HR-MS identification of AME-dihexoside (presumably AME-3-dihexoside, peak #AME-4). Comparison of the experimental (top) and theoretical (bottom) isotopic patterns. The structure is putative, actual positions may vary.

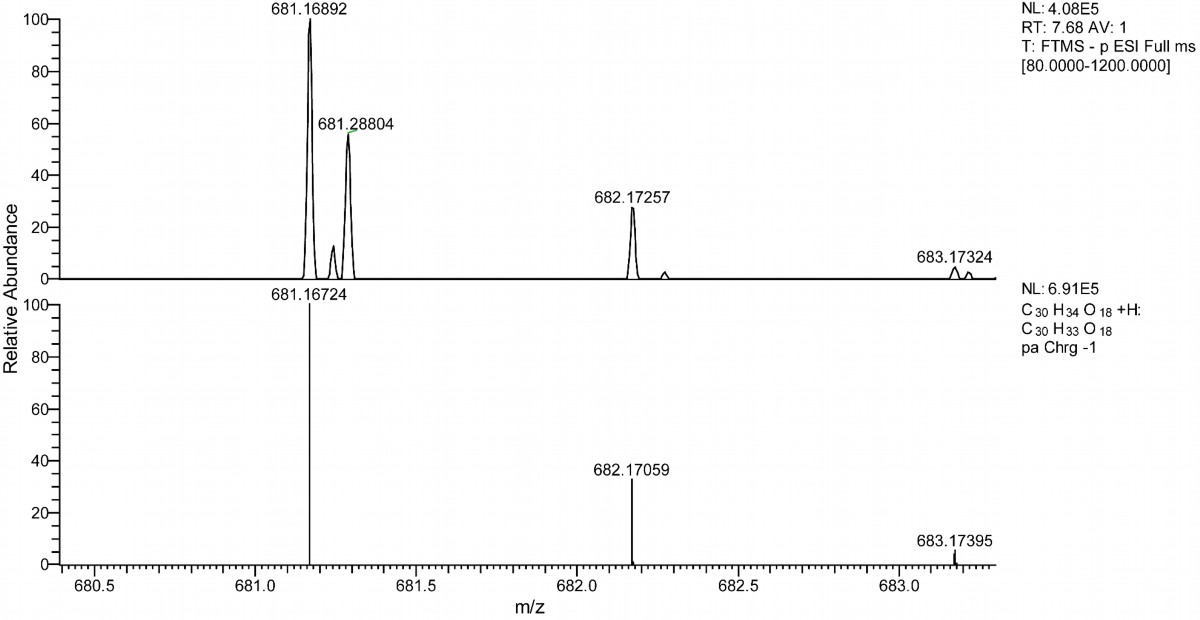


**Fig. S47.** HR-MS identification of AME-malonyldihexoside (presumably AME-7-hexosyl-3-malonylhexoside, peak #AME-6). Comparison of the experimental (top) and theoretical (bottom) isotopic patterns. The structure is putative, actual positions may vary.

**a)**

**b)**


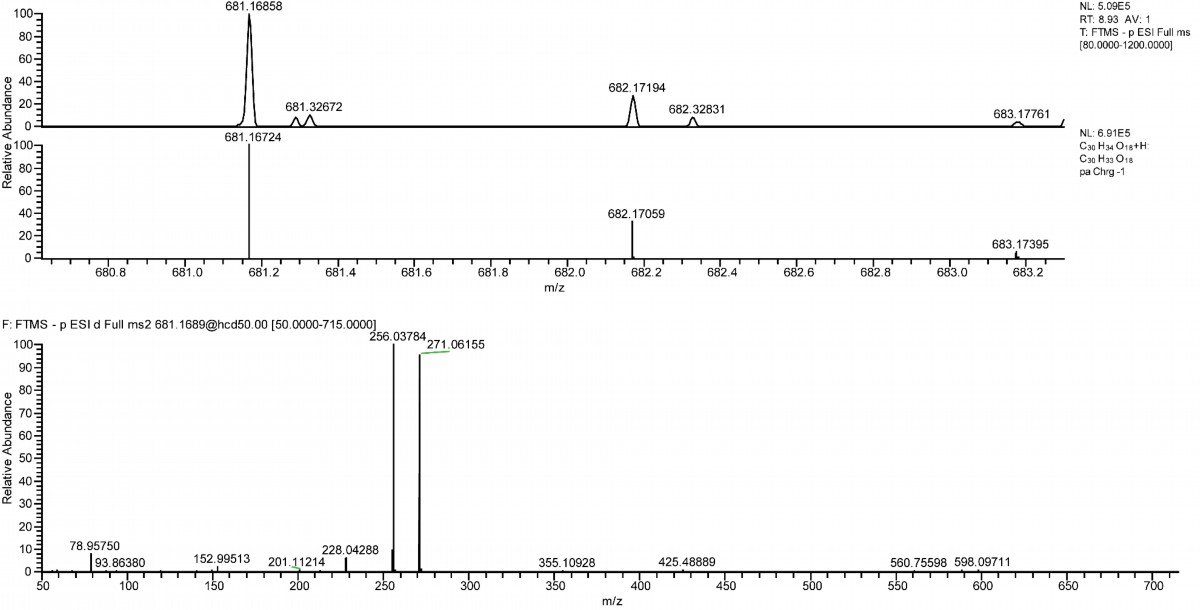


**Fig. S48.** HR-MS identification of AME-malonyldihexoside (presumably AME-7-malonyldihexoside, peak #AME-11). a) Comparison of the experimental (top) and theoretical (bottom) isotopic patterns. b) MS^2^ spectrum. The structure is putative, actual positions may vary.

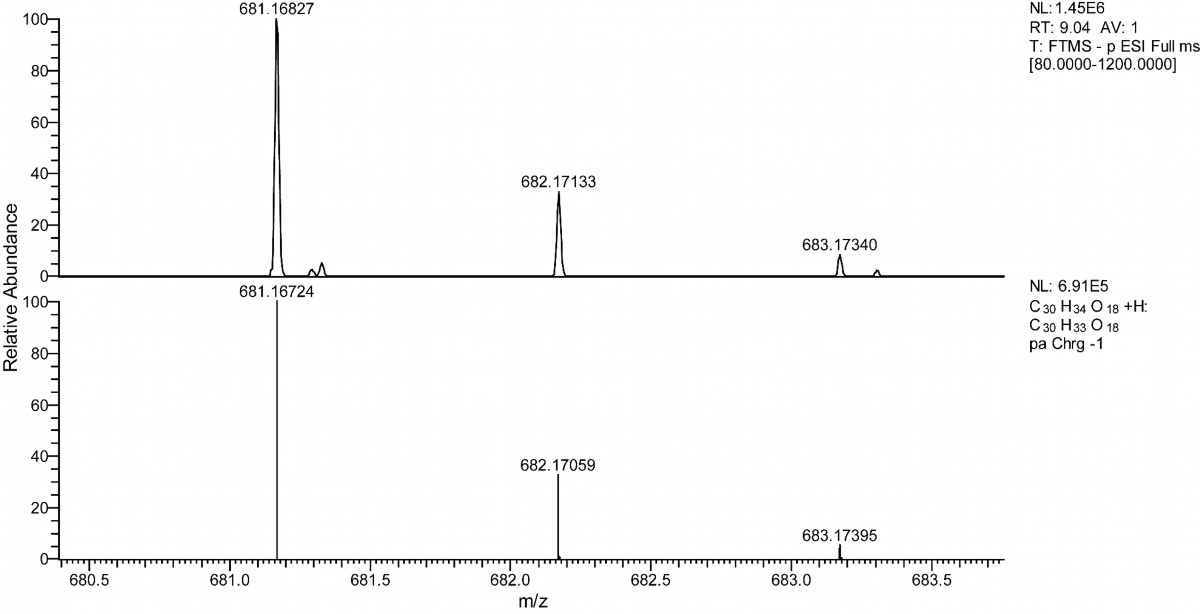


**Fig. S49.** HR-MS identification of AME-malonyldihexoside (presumably AME-3-malonyldihexoside, peak #AME-12). Comparison of the experimental (top) and theoretical (bottom) isotopic patterns. The structure is putative, actual positions may vary.

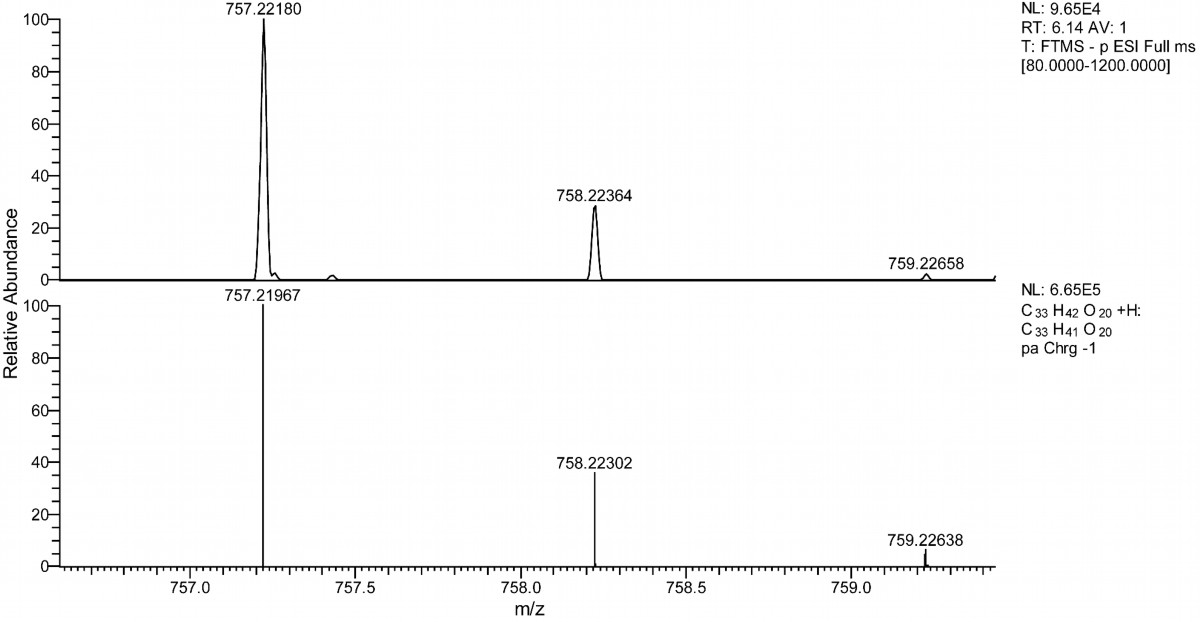


**Fig. S50.** HR-MS identification of AME-trihexoside (presumably AME-3-hexosyl-7-dihexoside, peak #AME-1). Comparison of the experimental (top) and theoretical (bottom) isotopic patterns. The structure is putative, actual positions may vary.

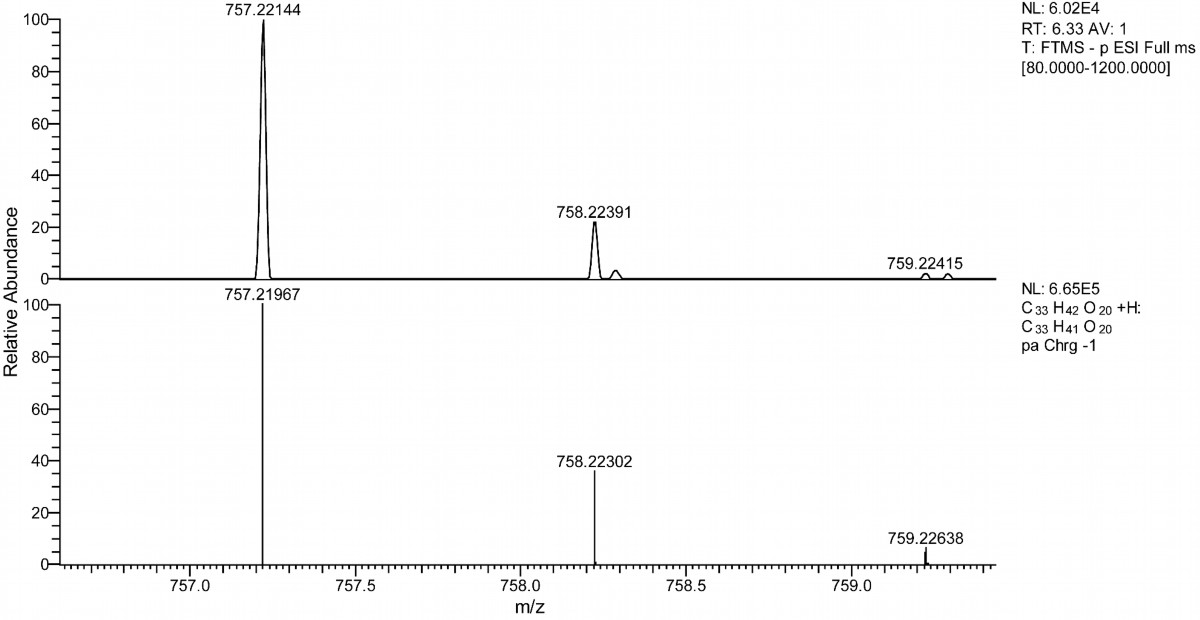


**Fig. S51.** HR-MS identification of AME-trihexoside (presumably AME-7-hexosyl-3-dihexoside, peak #AME-3). Comparison of the experimental (top) and theoretical (bottom) isotopic patterns. The structure is putative, actual positions may vary.

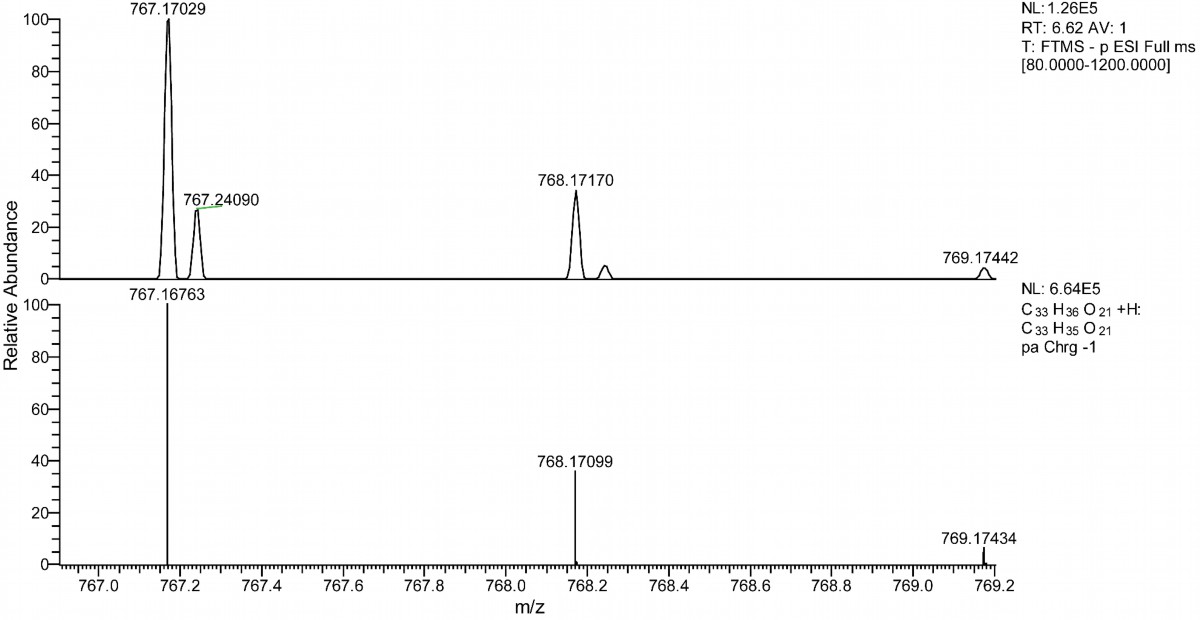


**Fig. S52.** HR-MS identification of AME-dimalonyldihexoside (presumably AME-3,7-dimalonyldihexoside, peak #AME-5). Comparison of the experimental (top) and theoretical (bottom) isotopic patterns. The structure is putative, actual positions may vary.

1. § Current address: German Federal Office of Consumer Protection and Food Safety, Diedersdorfer Weg 1, 12277 Berlin [↑](#footnote-ref-1)
